# Progressive HNF1A-MODY pathophysiology revealed by a translational mouse model

**DOI:** 10.1172/jci.insight.198095

**Published:** 2026-05-08

**Authors:** Isaline Louvet, Ana Acosta-Montalvo, Chiara Saponaro, Maria Moreno-Lopez, Sana Douffi, Abdelkrim El Karchaoui, Gianni Pasquetti, Julien Thevenet, Nathalie Delalleau, Valery Gmyr, Paolo Giacobini, Stéphanie Espiard, Julie Kerr-Conte, François Pattou, Adrian Liston, Caroline Bonner

**Affiliations:** 1 University of Lille, CHU Lille, Inserm U1190, European Genomic Institute for Diabetes, Institut Pasteur de Lille, Lille, France.; 2 Laboratory of Development and Plasticity of the Neuroendocrine Brain, FHU 1000 Days for Health, School of Medicine, Lille, France.; 3 University of Lille, Inserm, CHU Lille, Lille Neuroscience & Cognition, UMR-S 1172, Lille, France.; 4 Department of Pathology, University of Cambridge, Cambridge, United Kingdom.

**Keywords:** Aging, Endocrinology, Metabolism, Beta cells, Diabetes

## Abstract

HNF1A-MODY, the most common monogenic diabetes, exhibits progressive β cell dysfunction, but existing mouse models fail to recapitulate human disease progression, limiting understanding of pathogenic mechanisms. We developed mice with heterozygous deletion of the *Hnf1a* transactivation domain (*Hnf1a*^+/*Δ*e4-10^) to model human *HNF1A* haploinsufficiency, conducted cross-sectional metabolic characterization, and validated our findings in HNF1A-deficient human islets. Unlike previous models, *Hnf1a*^+/*Δ*e4-10^ mice successfully recapitulated temporal HNF1A-MODY progression. Male mice developed sequential pathophysiology: early insulin resistance in young adults (7 weeks), followed by testosterone deficiency and fasting hyperglycemia in adult mice (10 weeks). Glucose intolerance emerged in middle-aged mice (30 weeks), progressing to multi-organ dysfunction in aged mice (44–70 weeks), characterized by elevated hepatic gluconeogenesis, impaired renal glucose handling, and hepatic steatosis/fibrosis. This dual pathophysiology involving β cell dysfunction and peripheral insulin resistance was associated with dysregulated hormone secretion from both α and β cells in aged mice (40–70 weeks). Human islet studies with *HNF1A* knockdown confirmed translational relevance, demonstrating reduced SGLT2 protein expression and inappropriate glucagon and insulin secretion. This work established a physiologically relevant HNF1A-MODY model, identified early insulin resistance as a key mechanism triggering hormonal dysfunction, and revealed HNF1A’s role in multi-organ pathophysiology beyond traditional β cell dysfunction.

## Abstract 2

New mouse model reveals HNF1A-MODY diabetes involves early insulin resistance and testosterone deficiency before β-cell failure, affecting multiple organs beyond the pancreas.

## Graphical abstract

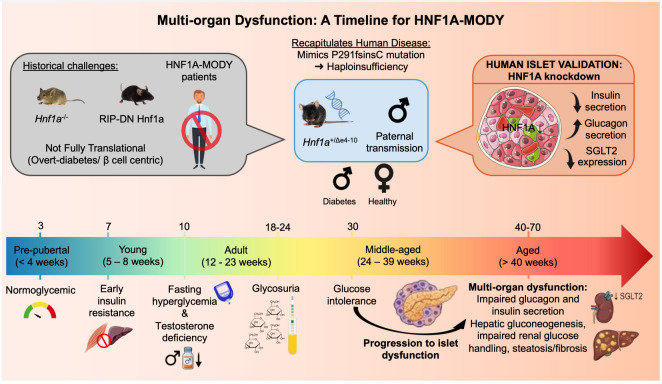

## Introduction

HNF1A-MODY affects approximately 1 in 10,000 individuals with diabetes and represents a critical model for understanding progressive β cell dysfunction in monogenic disease (1–3). Unlike type 1 diabetes (T1D) with its acute onset or type 2 diabetes (T2D) with its complex polygenic etiology, HNF1A-MODY offers unique insights into how single-gene defects drive predictable metabolic deterioration. The transcription factor hepatocyte nuclear factor-1α (HNF1A) orchestrates a complex network of genes essential for glucose homeostasis across multiple metabolic tissues (1, 4). Initially identified as a liver-enriched transcription factor, HNF1A has emerged as a master regulator of glucose metabolism (4, 5). Heterozygous pathogenic variants in *HNF1A* cause the most common form of monogenic diabetes, with over 1,000 distinct pathogenic variants identified (6–9). Beyond monogenic diabetes, *HNF1A*’s significance in glucose homeostasis is further highlighted by genome-wide association studies that have identified common variants in this gene as susceptibility loci for T2D across diverse populations (10–12).

Current understanding of HNF1A-MODY progression is limited by the lack of predictive biomarkers for disease onset and progression rates. While patients typically develop diabetes in young adulthood, the specific triggers and temporal sequence of organ dysfunction remain unclear (11, 12). The characteristic onset during periods of hormonal transition suggests potential endocrine influences that have not been systematically investigated. Mutation type and location influence both age of onset and disease progression, with mutations affecting the DNA-binding domain or causing frameshifts typically manifesting with more severe phenotypes compared with missense mutations in the transactivation domain (9). While HNF1A-MODY is classically characterized by β cell dysfunction, emerging evidence indicates that *HNF1A* mutations can also drive insulin resistance. Notably, the HNF1A-G319S mutation, identified in the Oji-Cree population, is associated with adult-onset insulin-resistant, obesity-related diabetes rather than classical MODY (13, 14). This mutation accelerates diabetes onset by approximately 7 years per allele, demonstrating that HNF1A deficiency can manifest through insulin resistance pathways in addition to primary β cell dysfunction. Understanding these mechanisms is crucial for developing predictive models and identifying patients at risk for rapid disease progression, particularly given the variable expressivity observed among carriers of identical *HNF1A* mutations (9, 15).

Previous attempts to model HNF1A-MODY have yielded inconsistent phenotypes that fail to recapitulate human disease progression. Conventional knockout models (*Hnf1a^–/–^*) develop severe diabetes shortly after weaning, while heterozygous *Hnf1a^+/–^* mice paradoxically maintain normal glycemia throughout life (16–20). Similarly, conditional β cell–specific *Hnf1a*-knockout models require homozygous mutation to display hyperglycemia, with heterozygotes remaining normoglycemic (21). These findings contrast sharply with human HNF1A-MODY, in which heterozygous mutations are sufficient to cause progressive diabetes. Furthermore, β cell–specific models cannot capture the systemic effects of *Hnf1a* dysfunction across multiple metabolic tissues (22). Importantly, these previous models have failed to capture the dual pathophysiology that characterizes human HNF1A-MODY, both β cell dysfunction and peripheral insulin resistance. Clinical studies demonstrate that secondary insulin resistance contributes to chronic hyperglycemia in MODY3 patients (23). Recent stem cell–derived models have provided valuable cellular insights but cannot replicate the complex in vivo pathophysiological progression and organ crosstalk that characterize human disease (24, 25).

Emerging clinical evidence reveals complex bidirectional interactions between metabolic dysfunction and sex hormone regulation in diabetes pathogenesis. In men, low testosterone levels increase T2D risk, while conversely, insulin resistance and hyperglycemia can suppress testosterone production through hypothalamic-pituitary-gonadal axis dysfunction (26–30). Mechanistically, testosterone enhances insulin sensitivity in skeletal muscle and modulates hepatic glucose production, while insulin resistance can impair gonadal steroid synthesis (31, 32). Recent work by Xu et al. demonstrated that testosterone and androgen receptor signaling in β cells are critical for normal glucose homeostasis specifically in males, with testosterone activating extranuclear androgen receptors to amplify GLP1 insulinotropic action (33). Given that HNF1A-MODY typically manifests during young adulthood and emerging evidence suggests that *HNF1A* deficiency affects insulin signaling pathways, investigating the temporal relationship between early metabolic dysfunction and subsequent hormonal changes could provide crucial insights into disease progression mechanisms. Understanding these connections could identify new biomarkers and disease modifiers, which would be particularly relevant given the growing recognition of hormonal factors in metabolic disease.

To address these knowledge gaps, we engineered a refined mouse model that achieves true *HNF1A* haploinsufficiency and recapitulates human disease progression. Using Cre-lox recombination, we deleted the genomic region encoding the transactivation domain within the native *Hnf1a* gene while preserving the dimerization and DNA-binding domains. This approach recapitulates the functional consequences of pathogenic human mutations that produce truncated proteins lacking transcriptional activity (34). Our comprehensive approach, combining longitudinal mouse studies with human islet validation, reveals early insulin resistance as a key triggering mechanism that initiates a pathophysiological cascade including hormonal dysfunction and progressive multi-organ deterioration. Complementary studies in *HNF1A*-deficient human islets revealed previously unknown roles of *HNF1A* in α cell regulation, uncovering mechanisms underlying abnormal glucagon secretion. Collectively, our work establishes a physiologically relevant model of HNF1A-MODY progression and provides a comprehensive framework for understanding the complex interplay between metabolic dysfunction, hormonal dysregulation, and multi-organ deterioration that characterizes this monogenic diabetes. These findings identify potential biomarkers for disease monitoring and provide a foundation for understanding individual variation in disease progression.

## Results

Current mouse models inadequately recapitulate the progressive pathophysiology of human HNF1A-MODY, limiting mechanistic understanding and therapeutic development. Complete knockout models (*Hnf1a^–/–^*) develop severe diabetes shortly after weaning, while conventional heterozygous *Hnf1a^+/–^* mice paradoxically maintain normal glycemia throughout life (16). To overcome these limitations and model the gradual metabolic deterioration characteristic of human HNF1A-MODY, we engineered a refined mouse model that precisely mimics human *HNF1A* haploinsufficiency.

**Figure 1:**
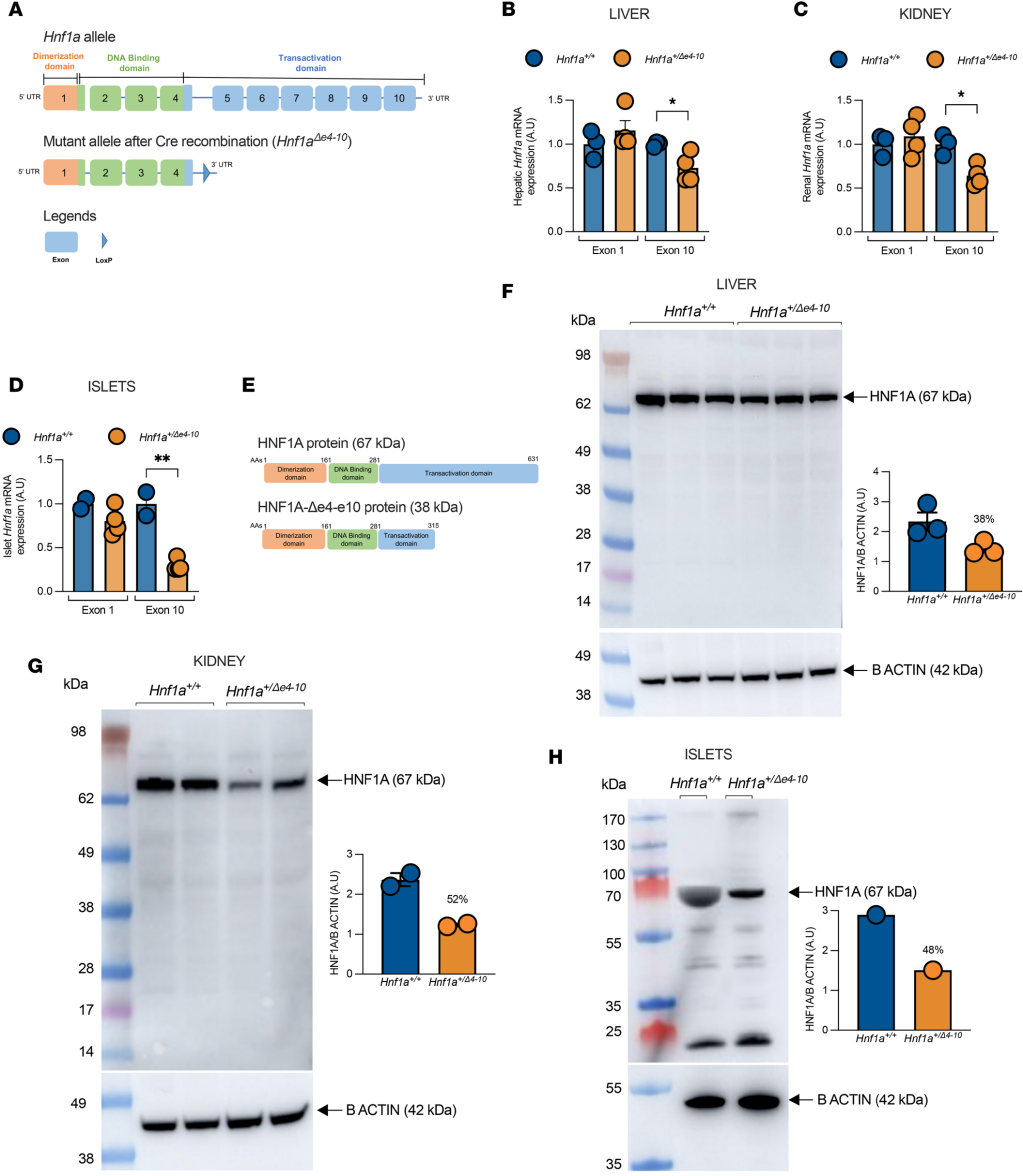
Generation and characterization of *Hnf1a^+/Δe4-10^* mice. (**A**) Structure of the wild-type *Hnf1a* gene, and the mutant Δe4-10 allele after Cre recombination. (**B**–**D**) *Hnf1a* gene expression analyzed by real-time PCR using primers targeting exon 1 and exon 10 in *Hnf1a^+/Δe4-10^* mice compared with *Hnf1a^+/+^* sibling controls in liver (**B**), kidney (*n =* 3–4 mice per group) (**C**), and isolated islets (pooled from *n =* 5 mice per group) (**D**). Gene expression was normalized to *Rpl27* mRNA. (**E**) Schematic representation of the wild-type HNF1A protein (67 kDa) and the predicted truncated HNF1A-Δe4-10 protein (38 kDa). (**F**–**H**) Western blot analysis of HNF1A protein from liver (**F**), kidney (**G**), and islets (**H**) showing reduced full-length HNF1A protein (67 kDa) in mutant compared with *Hnf1a^+/+^* sibling controls. Protein lysates (35 μg liver/kidney and 20 μg islets) were loaded with β-actin as loading control. Data are expressed as means ± SEM. *P < 0.05, **P < 0.01 by unpaired *t* test.

Using Cre-lox recombination, we strategically deleted the genomic region encoding the transactivation domain within the native *Hnf1a* gene while preserving both the dimerization and DNA-binding domains (Figure 1A and Supplemental Figure 1A; supplemental material available online with this article; https://doi.org/10.1172/jci.insight.198095DS1). This approach closely recapitulates the functional consequences of pathogenic human mutations, which often result in truncated proteins lacking transcriptional activity (34). Following a systematic breeding strategy, we first crossed *Hnf1a^loxP/loxP^* mice with CMV-Cre transgenic mice to achieve global expression of the Hnf1aΔe4-10 allele. Crucially, the metabolic phenotype displayed a strict parent-of-origin effect, manifesting exclusively upon paternal transmission of the Hnf1aΔe4-10 allele (Supplemental Figure 1B). Accordingly, we utilized this paternal inheritance pattern for our breeding strategy, which simultaneously eliminated the Cre transgene and generated experimental cohorts alongside littermate controls for all subsequent analyses. To ensure genetic consistency and validate our model, we implemented a rigorous genotyping protocol with dual PCR assays. The first assay confirmed the presence of loxP sequences (Supplemental Figure 1C), while the second specifically detected the constitutive Δe4-10 allele resulting from successful recombination (Supplemental Figure 1D). We selected only mice with confirmed efficient Cre-mediated recombination for subsequent breeding. Recognizing the potential impact of genetic background on metabolic phenotypes, we carefully controlled for confounding factors throughout our breeding strategy. In particular, we standardized the status of the nicotinamide nucleotide transhydrogenase (*Nnt*) gene (Supplemental Figure 1B), which is known to influence glucose homeostasis in certain mouse strains and could potentially mask or exaggerate phenotypes if not properly controlled (35, 36).

Molecular characterization confirmed successful achievement of haploinsufficiency. qPCR analysis using primers specific to exon 1 revealed comparable *Hnf1a* expression levels between *Hnf1a*^+/*Δ*e4-10^ and wild-type (*Hnf1a^+/+^*) mice in liver, kidney, and isolated islets (Figure 1, B–D), confirming normal transcription initiation from both alleles. In contrast, primers targeting exon 10 detected significant transcript reductions of 35% in liver (Figure 1B), 40% in kidney (Figure 1C), and 70% in isolated islets (Figure 1D) in *Hnf1a*^+/*Δ*e4-10^ mice compared with *Hnf1a^+/+^* siblings. This pattern confirms successful deletion of exons 4–10 in the mutant allele while maintaining normal expression of the wild-type allele, achieving true haploinsufficiency.

To assess the protein consequences of our genetic modification, we compared the predicted protein structure (Figure 1E) with actual protein expression (Figure 1, F–H). Western blot analysis demonstrated the functional impact, with significant reductions in full-length HNF1A protein (67 kDa) across all tissues: 38% in liver (Figure 1F), 52% in kidney (Figure 1G), and 48% in isolated islets (Figure 1H) of *Hnf1a*^+/*Δ*e4-10^ mice compared with *Hnf1a^+/+^* controls.

In pancreatic islets, we observed additional protein species migrating near the full-length band. Since rodents exclusively express the HNF1A-A isoform and lack the B and C isoforms found in humans (37), and these bands were present in both wild-type and mutant lysates, they likely represent endogenous posttranslational modifications rather than alternative isoforms or mutant products (Figure 1H). Crucially, despite detecting transcripts from the mutant allele, we observed no evidence of the predicted truncated HNF1A-Δe4-10 protein (38 kDa) in any tissue. This suggests that any truncated product is highly unstable and is likely cleared via nonsense-mediated decay or proteasomal degradation. These findings confirm that our model achieves true haploinsufficiency through reduced expression of functional full-length protein without generating a potentially interfering truncated variant, closely mirroring the molecular pathophysiology observed in human HNF1A-MODY patients with frameshift or nonsense mutations (38).

**Figure 2:**
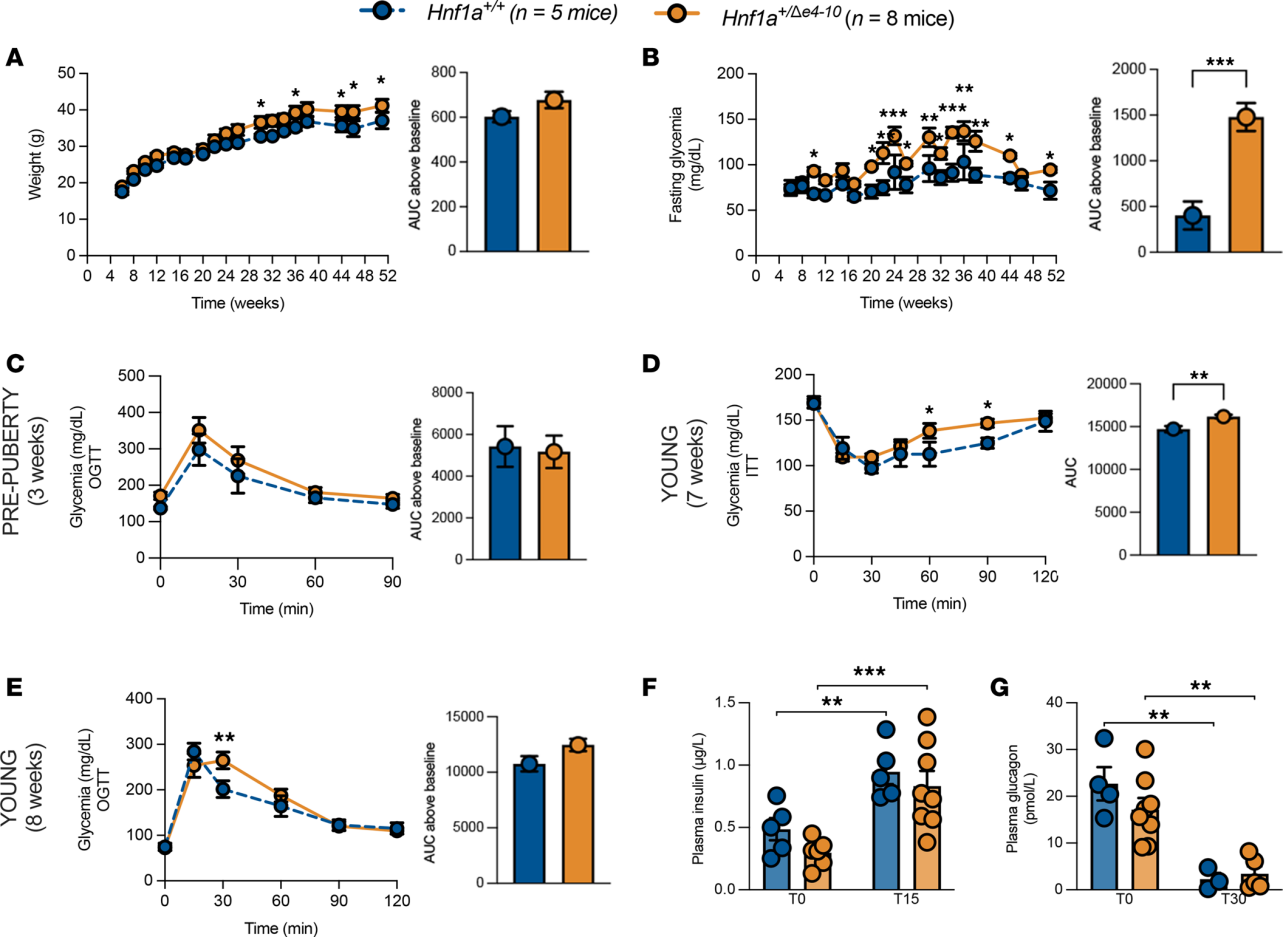
Metabolic characterization of young *Hnf1a^+/Δe4-10^* male mice. (**A** and **B**) Body weight (**A**) and fasting glycemia (**B**) with AUC after 16-hour overnight fast from 6 to 52 weeks of age. (**C**) Pre-pubertal mice (3 weeks): OGTT (2 g/kg) with AUC. (**D**) Insulin tolerance test (ITT) at 7 weeks with AUC. (**E**–**G**) Young mice (6–8 weeks): OGTT (2 g/kg) with AUC (**E**), plasma insulin (fasting and 15 minutes after glucose) (**F**), and plasma glucagon (fasting and 30 minutes after glucose) (**G**). Data are expressed as means ± SEM (*n =* 5–8 mice per group). *P < 0.05, **P < 0.01, ***P < 0.001 by 2-way ANOVA with mixed-effects analysis for longitudinal data or unpaired *t* test for single comparisons.

After confirming that our *Hnf1a*^+/*Δ*e4-10^ model achieves true haploinsufficiency, we conducted comprehensive cross-sectional metabolic phenotyping to determine whether it recapitulates the progressive nature of HNF1A-MODY. Based on established developmental milestones in mice (39), we stratified our analyses into distinct age groups: (a) pre-puberty (3 weeks); (b) young (6–7 weeks); (c) adult (8–23 weeks); (d) middle-aged (24–40 weeks); and (e) aged (>40 weeks) (21). Initial studies revealed that male, but not female (Supplemental Figure 2 and Supplemental Figure 3), *Hnf1a*^+/*Δ*e4-10^ mice developed age-dependent metabolic abnormalities, leading us to focus detailed characterization on males. Both genotypes showed comparable weight gain throughout development, with mutants exhibiting a slight but significant increase in late adulthood (Figure 2A). Notably, *Hnf1a*^+/*Δ*e4-10^ mice displayed characteristic temporal progression of metabolic dysfunction, with fasting hyperglycemia emerging at young age (10 weeks) and persisting with age (>50 weeks) (Figure 2B).

To establish the temporal sequence of this metabolic dysfunction, we first assessed glucose homeostasis in pre-pubertal mice. At 3 weeks of age, oral glucose tolerance was comparable between mutant and control mice (Figure 2C), indicating that glucose handling is preserved early in life. However, metabolic defects emerged shortly thereafter; *Hnf1a*^+/*Δ*e4-10^ mice exhibited significant insulin resistance as early as 7 weeks of age, characterized by impaired glucose disposal following insulin administration (Figure 2D). Consistent with this, oral glucose tolerance testing (OGTT) revealed a significant transient glucose elevation at 30 minutes (Figure 2E). Despite a similar overall AUC, this transient peak is physiologically significant as it reveals early-stage insulin resistance that precedes overt β cell failure. Collectively, these data identify insulin resistance as the earliest detectable metabolic abnormality in our model, occurring well before the development of fasting hyperglycemia.

**Figure 3:**
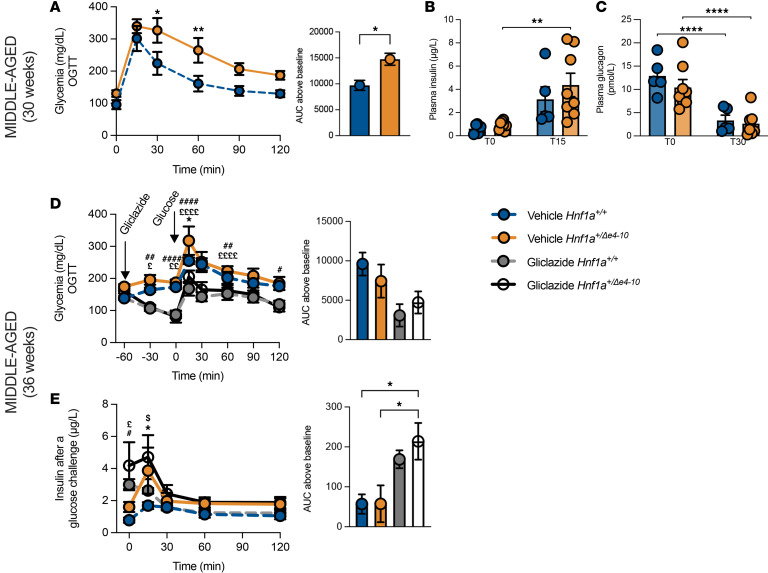
Metabolic characterization of middle-aged *Hnf1a^+/Δe4-10^* male mice. Middle-aged mice (24–40 weeks): (**A**–**C**) OGTT with AUC (**A**), plasma insulin (**B**), and plasma glucagon (**C**). (**D** and **E**) Sulfonylurea responsiveness (36 weeks): Glycemia during OGTT with gliclazide (10 mg/kg) or vehicle pretreatment (**D**) and insulin levels (**E**). Data are expressed as means ± SEM (*n =* 5–8 mice per group). *P < 0.05, **P < 0.01, ****P < 0.0001 by 2-way ANOVA with mixed-effects analysis for longitudinal data or unpaired *t* test for single comparisons. For sulfonylurea studies: * and $ indicate comparison between genotypes within each treatment (vehicle and gliclazide, respectively); £ and # indicate comparison between treatments within each genotype (*Hnf1a^+/+^* and *Hnf1a^+/Δe4-10^*, respectively).

Despite the underlying insulin resistance, young *Hnf1a*^+/*Δ*e4-10^ mice maintained glucose tolerance, with plasma insulin and glucagon profiles indistinguishable from controls (Figure 2, F and G). This suggests that compensatory mechanisms initially preserve glucose homeostasis in early adulthood. However, this stability was transient; by middle age (30 weeks), mutants developed significant glucose intolerance (Figure 3A). Notably, this metabolic deterioration occurred despite maintained plasma insulin and glucagon levels at both fasting and post–glucose challenge time points (Figure 3, B and C), indicating a growing disconnect between glycemic control and circulating hormone levels.

To validate the translational relevance of our model, we assessed β cell responsiveness to sulfonylureas, the first-line therapy for HNF1A-MODY (40). As expected, vehicle-treated *Hnf1a*^+/*Δ*e4-10^ mice displayed glucose intolerance compared with controls; however, gliclazide administration effectively mitigated this hyperglycemia in both genotypes (Figure 3D). Notably, *Hnf1a*^+/*Δ*e4-10^ mice exhibited enhanced insulin release compared with controls under both vehicle and gliclazide conditions (Figure 3E). This likely reflects compensatory hyperinsulinemia driven by the underlying insulin resistance described earlier. Importantly, this robust responsiveness to sulfonylureas confirms that the downstream insulin secretory machinery remains functional despite impaired glucose sensing. This dissociation closely mirrors the clinical profile of HNF1A-MODY patients and further validates the therapeutic fidelity of our model.

**Figure 4:**
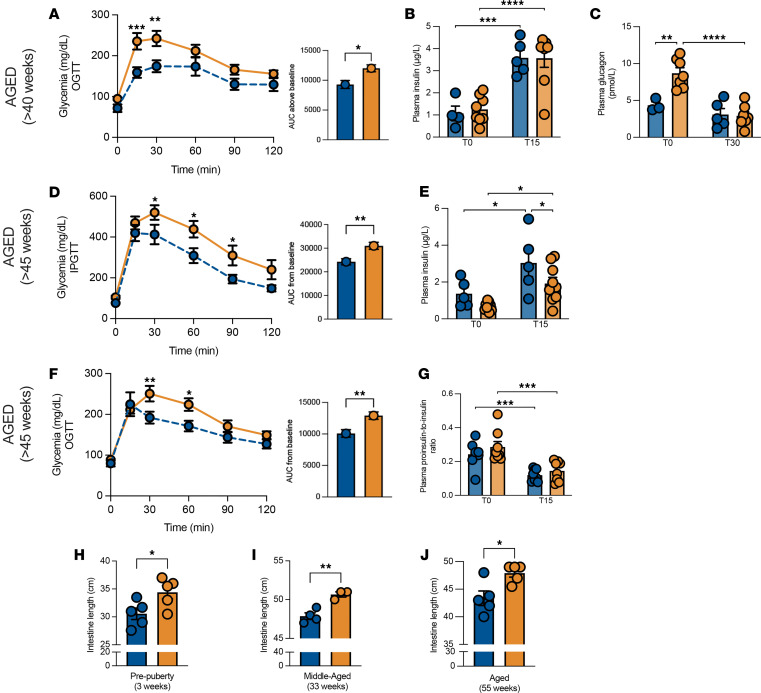
Metabolic characterization of aged *Hnf1a^+/Δe4-10^* male mice. (**A**–**G**) Aged mice (>40 weeks): OGTT with AUC (**A**), plasma insulin (**B**), and plasma glucagon (**C**); intraperitoneal glucose tolerance test (IPGTT) with AUC (**D**) and plasma insulin (**E**); and OGTT with AUC (**F**) and plasma proinsulin-to-insulin ratio (**G**). (**H**–**J**) Intestine length at 3 weeks (**H**), 33 weeks (**I**), and 55 weeks (**J**). Data are expressed as means ± SEM (*n =* 5–8 mice per group). *P < 0.05, **P < 0.01, ***P < 0.001, ****P < 0.0001 by 2-way ANOVA with mixed-effects analysis for longitudinal data or unpaired *t* test for single comparisons.

With age (>40 weeks), the metabolic phenotype deteriorated further. *Hnf1a*^+/*Δ*e4-10^ mice exhibited pronounced glucose intolerance (Figure 4A), while plasma insulin levels remained comparable to those in sibling controls (Figure 4B). Most notably, aged mutant mice displayed significantly elevated fasting glucagon levels (Figure 4C). This inappropriate hyperglucagonemia signals a distinct α cell dysfunction emerging late in disease progression, extending the pathophysiology beyond the traditional β cell–centric view of HNF1A-MODY.

To distinguish intrinsic β cell defects from gut-mediated effects, we performed intraperitoneal glucose tolerance tests in aged mice (>45 weeks) (Figure 4D). In contrast to the insulin response observed during oral challenge, intraperitoneal stimulation revealed a significant defect in acute insulin secretion at 15 minutes in *Hnf1a*^+/*Δ*e4-10^ mice compared with controls (Figure 4E). This secretory difference was not associated with impaired proinsulin processing, as the plasma proinsulin-to-insulin ratio decreased similarly in both genotypes following glucose stimulation (Figure 4, F and G). Parallel morphological characterization identified a potential compensatory driver: mutant mice exhibited significantly increased small intestine length compared with wild-type controls (Figure 4, H–J). This elongation was a constitutive phenotype, observed consistently from pre-puberty (3 weeks) through advanced age (55 weeks).

Mechanistically, we identified a disruption in the HNF1A regulatory network. Mutant islets showed reduced *Hnf4a* expression at both the mRNA (Supplemental Figure 4A) and protein levels (Supplemental Figure 4B). This was accompanied by the downregulation of key metabolic targets, including *Gck* (Supplemental Figure 4C), *Slc2a2* (Supplemental Figure 4D), and *Slc5a2* mRNA (Supplemental Figure 4E), alongside reduced SGLT2 protein (Supplemental Figure 4F). These findings suggest a hierarchical regulatory failure, consistent with our previous work establishing HNF1A as a direct regulator of *SLC5A2* expression in human islets (41).

Having established the progressive hormonal and glycemic abnormalities in *Hnf1a*^+/*Δ*e4-10^ male mice, we next investigated whether *Hnf1a* haploinsufficiency affects glucose homeostasis through extra-pancreatic mechanisms. This approach was prompted by *Hnf1a*’s well-documented roles in regulating both liver and kidney function (16, 42–46). We conducted a comprehensive analysis of these organs across different age groups to determine their contribution to the metabolic phenotype.

**Figure 5:**
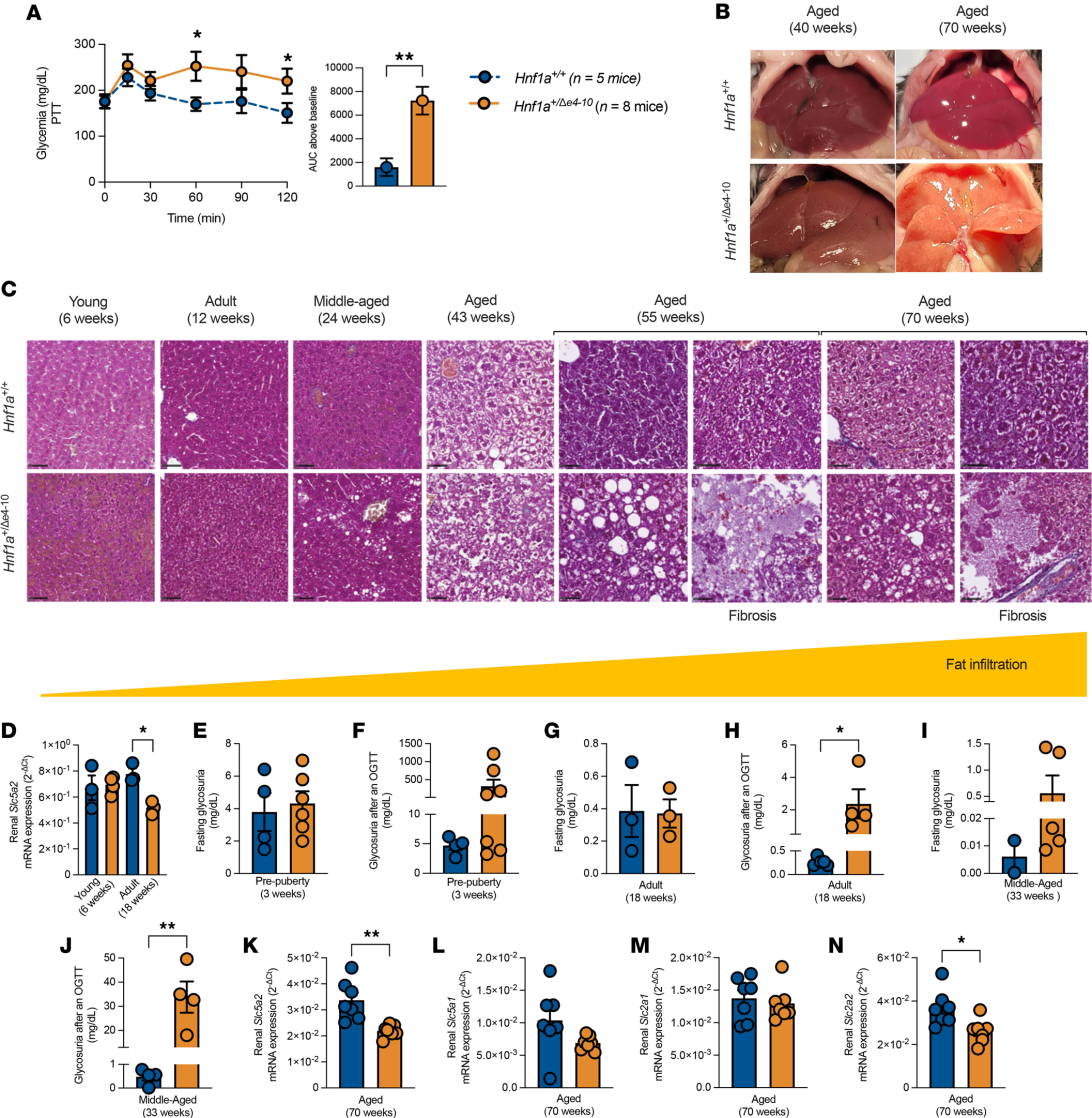
Hepatic and renal characteristics of *Hnf1a^+/Δe4-10^* mice. (**A**) Pyruvate tolerance test (2 g/kg) showing glycemic curve and AUC above baseline in 42-week-old mice after 6-hour fasting. (**B** and **C**) Temporal evolution of hepatic pathology with age. (**B**) Macroscopic liver appearance in *Hnf1a^+/+^* and *Hnf1a^+/Δe4-10^* mice at 40 weeks and 70 weeks. (**C**) Histological analysis with Masson’s trichrome staining in *Hnf1a^+/Δe4-10^* livers compared with *Hnf1a^+/+^* livers from 6 weeks to 70 weeks of age. Representative images at ×40 original magnification; scale bars: 50 μm. (**D**) RT-qPCR analysis of renal *Slc5a2* expression in young (6 weeks) and adult (18 weeks) mice. (**E**–**J**) Glycosuria measurements in pre-pubertal mice (3 weeks): fasting (**E**) and post-OGTT (**F**) glucose levels in urine; in adult mice (18 weeks): fasting (**G**) and post-OGTT (**H**) glucose levels in urine; and in middle-aged mice (30 weeks): fasting (**I**) and post-OGTT (**J**) glucose levels in urine. (**K**–**N**) RT-qPCR analysis of renal glucose transporters in aged mice (70 weeks): *Slc5a2* (**K**), *Slc5a1* (**L**), *Slc2a1* (**M**), and *Slc2a2* (**N**). Gene expression was normalized to *Rplp0* mRNA using the 2^–ΔCt^ method. Data are expressed as means ± SEM (*n =* mice 5–8 per group). *P < 0.05, **P < 0.01 by unpaired *t* test.

Pyruvate tolerance tests in aged (42 weeks) *Hnf1a*^+/*Δ*e4-10^ mice revealed significantly elevated glycemic excursions in comparison with *Hnf1a^+/+^* sibling controls (Figure 5A), with a 4.5-fold increase in area under the curve. This enhanced conversion of pyruvate to glucose demonstrates dysregulated hepatic gluconeogenesis. To determine whether this functional impairment was accompanied by structural alterations, we examined liver morphology across age. We observed progressive hepatic steatosis and fibrosis, which were evident macroscopically (Figure 5B) and histologically (Figure 5C) in aged mutants, confirming that HNF1A deficiency drives both functional gluconeogenic dysregulation and structural hepatopathy.

To elucidate the molecular drivers of this severe hepatic phenotype, we profiled key inflammatory and lipogenic pathways in aged livers (70 weeks). Consistent with the observed fibrosis, we detected a significant upregulation of proinflammatory markers, including *Il1b*, *Ccl2*, and *Ptgs2* (Supplemental Figure 5, A–F). Mechanistically, the steatotic phenotype appears to be driven by selective dysregulation of the carbohydrate-responsive pathway. We observed significantly increased expression of *Mixipl* (ChREBP) at the mRNA level (Supplemental Figure 5G) but no change in the protein level (Supplemental Figure 5H), leading to the upregulation of its downstream target, fatty acid synthase (FASN) (Supplemental Figure 5, I and J). Notably, the insulin-sensitive regulator *Srebf1* (SREBP1c) remained unchanged (Supplemental Figure 5, K and L). This suggests that the hepatic steatosis is driven specifically by ChREBP-mediated de novo lipogenesis, likely exacerbated by the prevailing hyperglycemia.

We next examined renal glucose handling, focusing on this tissue because HNF1A directly regulates sodium-glucose cotransporter-2 (SGLT2) in proximal tubule cells (16). qPCR analysis revealed a developmentally regulated loss of *Slc5a2* (SGLT2) expression: levels were preserved in young mice (6 weeks) but significantly reduced in adults (18 weeks) (Figure 5D), coinciding with the onset of systemic metabolic dysfunction.

This molecular defect had functional consequences across age. In pre-pubertal mice (3 weeks), fasting glycosuria was normal (Figure 5E), but mutants displayed a trend toward increased glucose excretion following an oral glucose challenge (Figure 5F). This pattern persisted into adulthood (18 weeks), where mutant mice again exhibited normal fasting glycosuria (Figure 5G) but significant glycosuria after glucose loading (Figure 5H). By middle age (33 weeks), the phenotype remained consistent, with preserved fasting glycosuria (Figure 5I) and elevated post-challenge excretion (Figure 5J), indicating impaired renal glucose reabsorption specifically under conditions of glycemic stress.

The renal phenotype continued to progress with age. By 70 weeks, *Hnf1a*^+/*Δ*e4-10^ mice showed a profound reduction in renal *Slc5a2* expression (Figure 5K). To determine whether this effect was specific to *Slc5a2* or represented a broader disruption of glucose transporters, we assessed additional targets. Expression of *Slc5a1* (SGLT1) trended lower in mutant mice but did not reach statistical significance (Figure 5L), while *Slc2a1* (GLUT1) was unchanged (Figure 5M). Notably, we observed significantly reduced *Slc2a2* (GLUT2) expression (Figure 5N), a finding that contrasts with previous reports showing no change in renal GLUT2 in homozygous *Hnf1a*-knockout mice (16).

A notable feature of our model was a marked sexual dimorphism; metabolic abnormalities were primarily evident in male mice, whereas females remained relatively protected. Given recent evidence that testosterone and androgen receptor (AR) signaling in β cells are critical for normal glucose homeostasis in males (33), we investigated whether HNF1A deficiency disrupts this hormonal axis.

**Figure 6:**
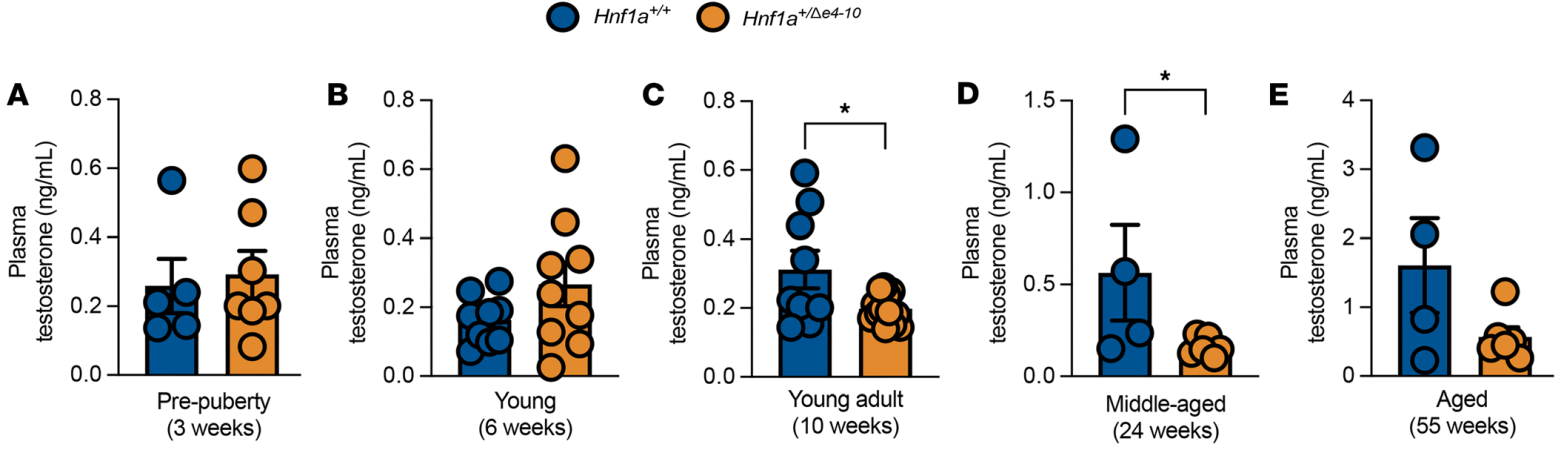
Testosterone levels in male *Hnf1a^+/Δe4-10^* mice. (**A**–**E**) Plasma testosterone levels in pre-pubertal mice (**A**), young mice (**B**), adult mice (**C**), middle-aged mice (**D**), and aged mice (**E**) (*n =* 4–9 per group). Data are presented as means ± SEM. *P < 0.05; unpaired *t* tests were used for statistical comparisons.

We first profiled circulating hormone levels. To control for diurnal and metabolic fluctuations, plasma testosterone was measured in the morning under fasted conditions (47). In pre-pubertal (3 weeks) and young mice (6 weeks), testosterone levels were comparable between genotypes (Figure 6, A and B). However, while control mice displayed the expected physiological rise in testosterone at puberty, mutant mice failed to mount this age-related increase, maintaining lower levels at 10 weeks (Figure 6C) and 24 weeks (Figure 6D), with this trend persisting in aged mice (55 weeks; Figure 6E). Downstream AR analysis, including AR protein expression in testis and islets (Supplemental Figure 6, A–C), islet *Ar* mRNA quantification (Supplemental Figure 6D), and hepatic *Ar* mRNA across the lifespan (Supplemental Figure 6, E–H), confirmed tissue-specific regulation.

Following the characterization of the systemic metabolic phenotype, marked by early insulin resistance and subsequent testosterone deficiency, we next investigated the intrinsic secretory capacity of *Hnf1a*^+/*Δ*e4-10^ islets. Immunofluorescence staining confirmed that HNF1A is expressed across major endocrine cell types, including insulin-positive β cells, glucagon-positive α cells, and somatostatin-positive δ cells, in both mouse and human islets (Supplemental Figure 7, A and B). Structurally, while gross islet morphology remained largely intact, aged mutant mice exhibited a significant expansion of the glucagon-positive compartment (Supplemental Figure 7C) accompanied by mild intra-pancreatic lipid infiltration (Supplemental Figure 7D). This widespread expression profile suggests that HNF1A haploinsufficiency likely compromises multiple hormone-secreting populations. To test this, we evaluated secretory dynamics using islet perifusion for insulin and static incubation for glucagon.

**Figure 7:**
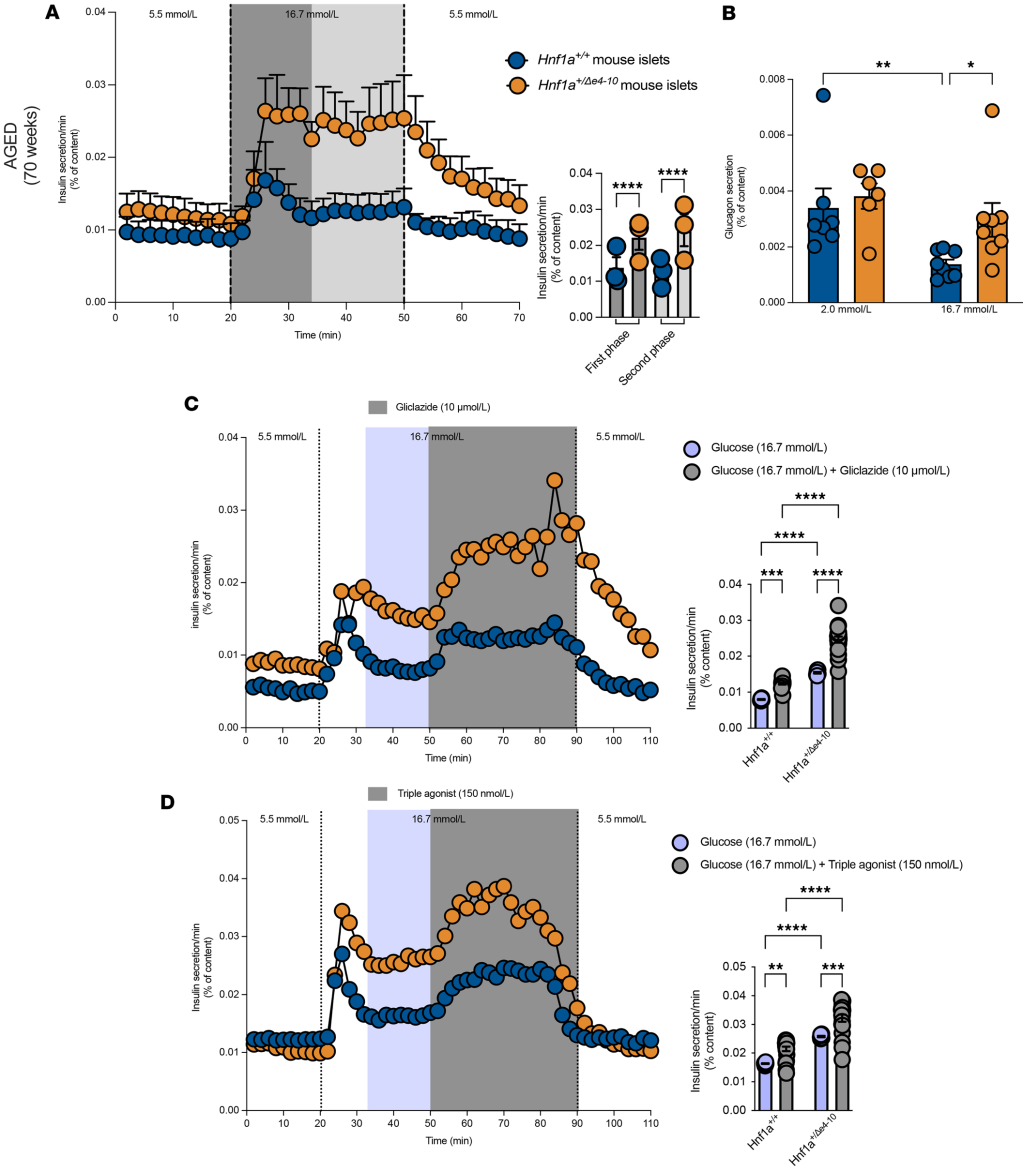
Insulin and glucagon secretion from isolated islets of *Hnf1a^+/+^* and *Hnf1a^+/Δe4-10^* mice. (**A**) Dynamic insulin secretion during perifusion with varying glucose concentrations (5.5 mmol/L → 16.7 mmol/L → 5.5 mmol/L) in islets isolated from *Hnf1a^+/+^* mice (blue) and *Hnf1a^+/Δe4-10^* mice (orange). Right: Quantification of first-phase and second-phase insulin secretion in response to high glucose. (**B**) Glucagon secretion during static incubation with varying glucose concentrations (2.0 mmol/L and 16.7 mmol/L) in islets from *Hnf1a^+/+^* and *Hnf1a^+/Δe4-10^* mice. (**C**) Dynamic insulin secretion in response to high glucose (16.7 mmol/L) with gliclazide (10 μmol/L) treatment in islets from aged mice (70 weeks). Right: Quantification of insulin secretion with glucose alone versus glucose plus gliclazide treatment. (**D**) Dynamic insulin secretion in response to high glucose (16.7 mmol/L) with triple agonist (150 nmol/L) treatment in islets from aged mice (70 weeks). Right: Quantification of insulin secretion with glucose alone versus glucose plus triple agonist treatment. Data are presented as means ± SEM. *P < 0.05, **P < 0.01, ***P < 0.001, ****P < 0.0001; statistical comparisons were performed using unpaired *t* tests.

Isolated islets from *Hnf1a*^+/*Δ*e4-10^ mice displayed markedly dysregulated glucose-stimulated insulin secretion (GSIS) compared with those from *Hnf1a^+/+^* sibling controls, characterized by elevated insulin secretion. Both first-phase and second-phase insulin secretion were significantly increased in mutant islets, indicating a hyperinsulinemic response relative to the normal response of controls (Figure 7A) (48, 49). We next examined α cell function by measuring glucagon secretion in response to glucose stimulation. Islets from *Hnf1a*^+/*Δ*e4-10^ mice showed comparable glucagon secretion at 2 mmol/L glucose compared with islets from *Hnf1a^+/+^* mice. However, unlike *Hnf1a^+/+^* islets, glucagon secretion was not suppressed in *Hnf1a*^+/*Δ*e4-10^ islets when glucose was increased to 16.7 mmol/L (Figure 7B). This dysregulated glucagon secretion pattern indicates that HNF1A deficiency disrupts the intrinsic glucose-sensing machinery of α cells, consistent with the elevated fasting glucagon levels observed in aged *Hnf1a*^+/*Δ*e4-10^ mice.

To validate that our model retains the defining therapeutic responsiveness of human HNF1A-MODY, we tested acute responsiveness to pharmacological agents. Given the clinical efficacy of sulfonylureas in HNF1A-MODY patients, we tested *Hnf1a*^+/*Δ*e4-10^ islet responses to gliclazide treatment. Mutant islets displayed enhanced sensitivity to sulfonylurea stimulation, with substantially greater incremental insulin responses in comparison with control islets (Figure 7C). This preserved and enhanced response to sulfonylureas suggests that insulin granule exocytosis machinery remains intact in *Hnf1a*^+/*Δ*e4-10^ β cells despite aberrant glucose sensitivity, confirming the clinical relevance of our model and providing a mechanistic explanation for the effectiveness of these agents in HNF1A-MODY patients.

To further characterize *Hnf1a*^+/*Δ*e4-10^ islet secretory capacity, we tested responses to incretin-based therapies. Treatment with a triple GLP1 receptor agonist, which simultaneously activates GLP1, GIP, and GCG receptors, enhanced insulin secretion in *Hnf1a*^+/*Δ*e4-10^ islets to levels comparable to those in treated control islets (Figure 7D). This preserved responsiveness to incretin-based agents demonstrates that multiple therapeutic pathways remain functional in our model.

**Figure 8:**
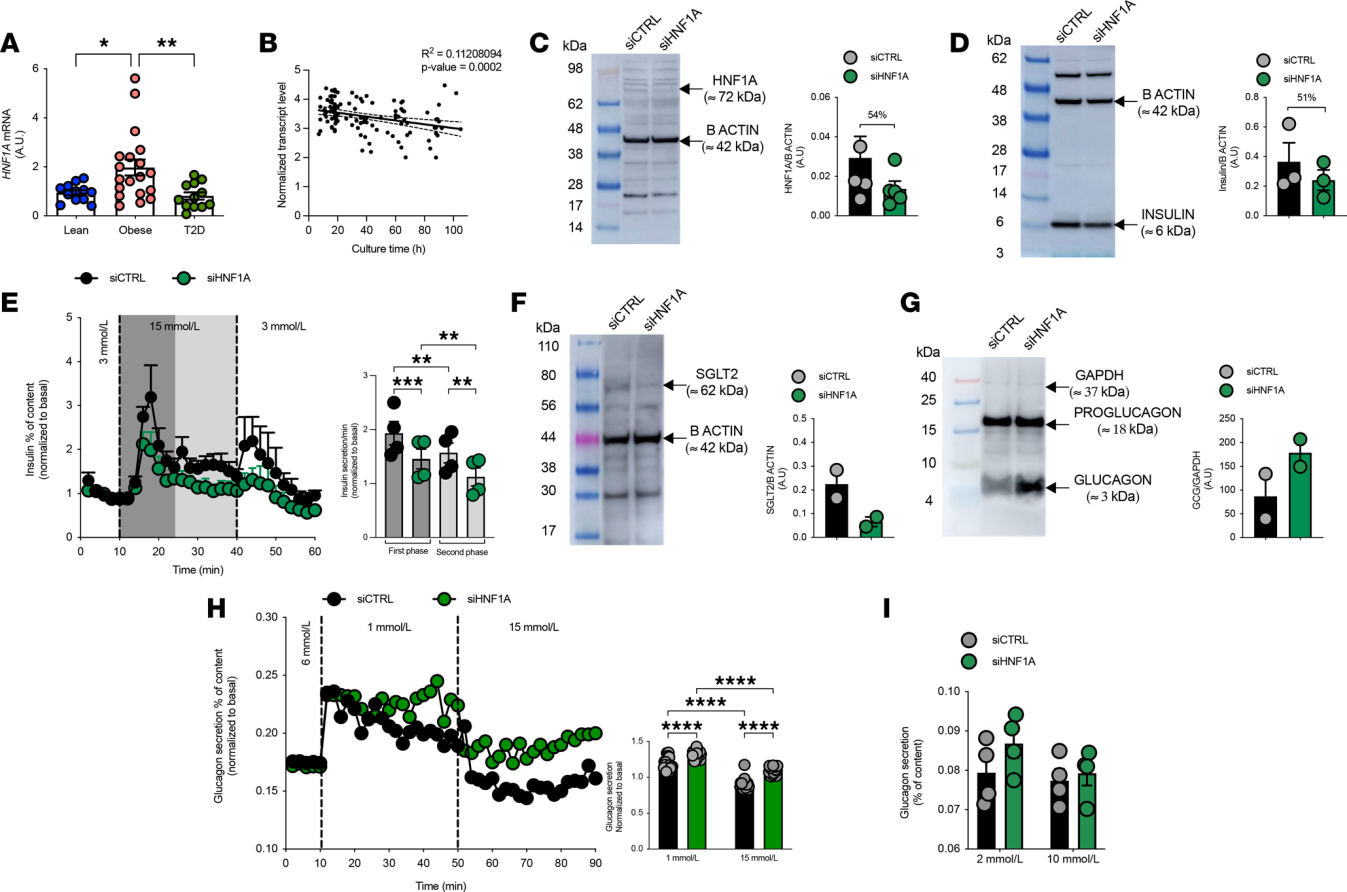
HNF1A knockdown in human islets recapitulates key features of *Hnf1a^+/Δe4-10^* mouse model. (**A**) *HNF1A* mRNA expression in human islets isolated from lean donors (*n =* 10), donors with obesity (*n =* 16), and T2D (*n =* 10) donors. (**B**) *HNF1A* transcript level in human islets over time in culture. (**C**) Western blot analysis and quantification of HNF1A protein after siRNA knockdown showing 54% reduction. (**D**) Western blot analysis and quantification of insulin protein after HNF1A knockdown showing 51% reduction. (**E**) Dynamic insulin secretion during glucose stimulation (3 mmol/L → 15 mmol/L → 3 mmol/L) in control (siCTRL, black) and siHNF1A (green) islets. Right: Quantification of first-phase and second-phase insulin secretion. (**F**) Western blot analysis and quantification of SGLT2 protein expression after HNF1A knockdown showing 70% reduction. (**G**) Western blot analysis of proglucagon and glucagon protein in control and HNF1A-deficient islets, with quantification showing 104% increase in glucagon after HNF1A knockdown. (**H**) Dynamic glucagon secretion in response to varying glucose concentrations (1 mmol/L → 15 mmol/L) with quantification at 1 mmol/L and 15 mmol/L glucose. (**I**) Static glucagon secretion at 2 mmol/L and 10 mmol/L glucose. Protein levels normalized to β-actin or GAPDH. Data are presented as means ± SEM. *P < 0.05, **P < 0.01, ***P < 0.001, ****P < 0.0001. One-way ANOVA with Tukey’s post hoc test was used for *HNF1A* mRNA comparisons, while unpaired *t* tests were used for protein quantifications and hormone secretion comparisons.

To translate our findings from the mouse model to human physiology and establish direct relevance to HNF1A-MODY, we investigated HNF1A function in isolated human pancreatic islets. First, we compared *HNF1A* gene expression across different metabolic conditions using human islets from separate donor groups. *HNF1A* mRNA was significantly higher in islets from donors with obesity compared with lean donors, while islets from T2D donors showed lower expression levels than islets from donors with obesity (Figure 8A). This expression pattern across different metabolic states suggests potential compensatory upregulation of *HNF1A* in obesity that appears absent in the diabetic state. Additionally, RNA-seq analysis revealed that *HNF1A* mRNA levels progressively decrease with time in culture (Figure 8B) (50), highlighting the importance of standardizing experimental conditions when studying this transcription factor.

To directly assess the functional consequences of *HNF1A* deficiency, we established a siRNA-mediated knockdown system in human islets. Transfection with *HNF1A*-targeted siRNA achieved a 54% reduction in HNF1A protein expression (Figure 8C), closely mirroring the haploinsufficiency observed in our mouse model. This knockdown led to a 51% reduction in insulin content (Figure 8D), consistent with *HNF1A*’s established role in regulating *INS* gene expression. Dynamic perifusion studies revealed impaired GSIS in *HNF1A*-deficient islets, with reduced responses to glucose stimulation (Figure 8E).

We next examined SGLT2 protein expression in human islets, as our group previously demonstrated that SGLT2 is specifically expressed in human α cells and that its inhibition, either by siRNAs targeting SGLT2 or by its selective pharmacological inhibitor, dapagliflozin, triggered glucagon secretion (41, 51, 52). This connection was particularly relevant given our observations of reduced renal *Slc5a2* expression and glycosuria in the *Hnf1a*^+/*Δ*e4-10^ mice. *HNF1A* knockdown led to a 70% reduction in SGLT2 protein levels in human islets (Figure 8F), with no changes in SGLT1, GLUT1, or GLUT2 (Supplemental Figure 8, A–C), confirming that HNF1A is a critical regulator of this glucose transporter.

The most notable effects of *HNF1A* knockdown, however, were observed in α cell function. While proglucagon protein content was not significantly altered, *HNF1A*-deficient human islets exhibited markedly dysregulated glucagon secretion (Figure 8, G–I). Similarly to our mouse model, *HNF1A* knockdown in human islets resulted in impaired glucagon suppression at high glucose concentrations and exaggerated glucagon secretion at low glucose levels. This finding highlights a previously underappreciated role for HNF1A in regulating α cell function and suggests that dysregulated glucagon secretion may be an important contributor to HNF1A-MODY pathophysiology.

## Discussion

We present a comprehensive characterization of a translational murine model of HNF1A-MODY that closely mirrors the human disease trajectory. Our study reveals a progressive metabolic syndrome characterized by a distinct temporal sequence: early insulin resistance, followed by fasting hyperglycemia, low testosterone levels, glycosuria, glucose intolerance, compensatory hyperinsulinemia, endogenous glucose production, and impaired glucagon and insulin release that evolve in a sex-specific manner. The development of a heterozygous *Hnf1a*^+/*Δ*e4-10^ mouse model that recapitulates the progressive nature of human HNF1A-MODY has enabled us to identify previously unrecognized pathogenic mechanisms with substantial implications for disease monitoring and biomarker development, offering a marked advantage over previous knockout models that display immediate, non-progressive severity (16, 17, 19, 46, 53).

A critical question in HNF1A-MODY research has been whether the disease is driven by haploinsufficiency or dominant-negative effects. While overexpressed truncated variants like p.P291fsinsC show dominant-negative properties in vitro, our in vivo data strongly support a haploinsufficiency model. We observed a consistent approximately 50% reduction in full-length HNF1A protein across islets, liver, and kidney, with a total absence of any detectable truncated 38 kDa product. This suggests that the mutant protein is highly unstable and cleared via degradation, aligning with clinical hypotheses that even dominant-negative mutations may function as loss-of-function alleles in vivo as a result of protein instability (54). The fact that a simple reduction in HNF1A protein is sufficient to drive progressive, multi-organ dysfunction provides a physiologically relevant framework to study the pathogenic threshold of HNF1A activity that is applicable to the broad spectrum of HNF1A-MODY patients.

Our most noteworthy finding is the identification of early insulin resistance as the initial metabolic defect that precedes and potentially triggers the pathophysiological cascade in HNF1A-MODY. This finding fundamentally reframes our understanding of disease pathogenesis, indicating that HNF1A-MODY involves dual pathophysiology encompassing both β cell dysfunction and peripheral insulin resistance from its earliest stages. The temporal precedence of insulin resistance is clinically relevant, as recent studies have demonstrated that secondary insulin resistance contributes to chronic hyperglycemia in HNF1A-MODY patients (23), and that HNF1A deficiency affects genes directly involved in insulin resistance pathways (25, 55). This concept aligns with clinical data from the Oji-Cree population, in which the HNF1A-G319S mutation is strongly linked to insulin-resistant, obesity-related diabetes rather than classical MODY, demonstrating that HNF1A deficiency can indeed manifest through insulin resistance pathways (13, 14). This early emergence of insulin resistance provides mechanistic insights into disease progression that purely β cell–focused models cannot capture and explains the progressive metabolic deterioration that characterizes human HNF1A-MODY.

The striking sexual dimorphism in our model, in which phenotypes manifest only in males, uncovered a potential link between HNF1A and the androgen signaling axis. We identified a metabolic-to-hormonal cascade where hepatic insulin resistance precedes lower testosterone levels, followed by a late-stage reduction in androgen receptor (AR) protein specifically within the islets. The absence of a comparable phenotype in female mutants contrasts with clinical observations in which HNF1A-MODY affects both sexes. While testosterone and AR levels have not been systematically evaluated in HNF1A-MODY patients, cistromic analysis revealed a key species difference: humans possess multiple AR binding sites near the *HNF1A* promoter that are absent in mice (Signaling Pathways Project; http://www.signalingpathways.org/index.jsf). This suggests that while AR may support *HNF1A* expression in humans, the male-specific severity in our mouse model is likely driven by a systemic collapse of androgen signaling acting as a “pathogenic amplifier” of the metabolic phenotype. Furthermore, the phenotype manifests specifically with paternal transmission in our model, suggesting an epigenetic or imprinting component that may be particularly dominant in the murine lineage. Additionally, the differential expression of AR, which is moderately expressed in the pancreas of male mice but absent in females (56), offers a compelling hypothesis for this sex-specific penetrance; females may be intrinsically protected simply because their islet function is less dependent on androgen signaling.

Our model further demonstrates that HNF1A-MODY is a systemic disorder involving coordinated multi-organ failure. We observed early post-OGTT glycosuria in pre-pubertal mice, recapitulating the lowered renal glucose threshold seen in HNF1A-MODY patients (57). Additionally, our findings validate the α cell dysfunction previously observed in models derived from human induced pluripotent stem cells and in HNF1A-MODY islets within a physiological in vivo context (25, 58–60). Mechanistically, HNF1A haploinsufficiency leads to a secondary downregulation of HNF4A, disrupting a critical transcriptional circuit that regulates shared target genes as demonstrated by Ferrer and colleagues (44). Given that SGLT2 inhibition stimulates glucagon secretion, the downregulation of SGLT2 in α cells provides a molecular mechanism for the hyperglucagonemia observed in our model and human HNF1A-MODY (41, 52, 59). Despite impaired glucose sensing, our model maintains preserved and often enhanced responsiveness to sulfonylureas and incretin-based therapies, validating the integrity of the fundamental secretory machinery. This clinical fidelity is reinforced by normal proinsulin-to-insulin ratios, indicating that proinsulin processing remains functional despite metabolic stress.

Several limitations of our study should be acknowledged. First, while our mouse model closely mimics the progressive pathophysiology of human HNF1A-MODY, notable species-specific differences exist. The striking male-specific phenotype in our mice contrasts with the equal sex distribution in human patients. Additionally, regarding hormonal assessment, we quantified total rather than free testosterone. While free testosterone is the gold standard for assessing bioactivity, particularly given the potential for altered sex hormone–binding globulin (SHBG) levels in liver disease, sample volume constraints precluded equilibrium dialysis. Complementary approaches using human stem cell models may have provided valuable insights, aligning with our observations in mouse and human islets. Despite these limitations, the convergence of phenotypes across our murine model and human islet data provides robust evidence for the mechanisms described.

By establishing what we believe to be the first physiologically relevant model of progressive HNF1A-MODY, these findings provide a foundation for developing personalized monitoring strategies and therapeutic interventions for patients with this common form of monogenic diabetes.

## Methods

### Sex as a biological variable

This study was designed to investigate HNF1A-MODY pathophysiology in both male and female mice, as well as human islets from both sexes. Initial screening revealed that metabolic abnormalities developed predominantly in male *Hnf1a*^+/*Δ*e4-10^ mice, while female mutants remained largely protected from metabolic dysfunction across the lifespan studied. Given this pronounced sexual dimorphism and the limited resources for comprehensive longitudinal studies, we focused detailed characterization on male mice to understand the mechanisms underlying disease progression. Human islet studies included donors of both sexes (Supplemental Table 1), and no sex-specific differences were observed in HNF1A expression or functional responses to knockdown.

### Animal husbandry and care

*Hnf1a^loxP/+^* mice were generated in C57BL/6JCya by Cyagen Bioscience. Female B6.C-Tg(CMV-Cre)1Cgn/J (strain 006054) mice were purchased from The Jackson Laboratory. Female C57BL/6NCrl mice were obtained from Charles River. *Hnf1a^loxP/loxP^* males were crossed with CMV-Cre females to generate *Hnf1a*^+/*Δ*e4-10^ founder males, which were then bred with wild-type C57BL/6NCrl females to produce experimental cohorts.

All mice were housed under specific pathogen–free conditions in a temperature-controlled room (21°C–22°C) with a 12-hour light/12-hour dark cycle and ad libitum access to food and water. All experiments were performed in male mice from 3 weeks to 70 weeks of age. Mice were maintained on a standard chow diet containing 26.2% protein, 52% carbohydrates, 4% fiber, 5.1% lipids, 5.4% vitamins and minerals, and 7.3% moisture (catalog A03, SAFE Diets, France). The total number of mice used was 126 (Supplemental Table 2).

### Genotyping

After weaning (22–23 days), mice were tagged, and a small piece of the tail was collected for genotyping. For DNA extraction, tails were incubated overnight at 55°C with 100 μL DirectPCR Lysis Reagent (Mouse Tail) (Viagen Biotech) supplemented with 1 μL of proteinase K (Agilent Dako) according to Viagen Biotech’s instructions. After overnight lysis, samples were incubated for 45 minutes at 85°C. DNA was stored at –20°C until genotyping. Genotyping protocol was performed for Cre-CMV, Hnf1aloxP/+, and Hnf1a-LoxP CMV-Cre mouse lines. A volume of 1 μL of extracted DNA was added to 19 μL of PCR mix containing 7 μL of DNase/RNase-Free Distilled Water (Thermo Fisher Scientific), 10 μL of PhireTaq Mix (Thermo Fisher Scientific), 0.5 μL of primer forward, and 0.5 μL of primer reverse in the case of PCR with 2 primers, or 1 μL of primer forward and 0.5 μL of primer reverse 1 and 0.5 μL of primer reverse 2 in the case of PCR with 3 primers. Primer sequences are listed in Supplemental Table 3. Primers were used in a 10 pmol/μL concentration. Target sequences were amplified using the following cycling conditions: initial denaturation at 94°C for 5 minutes, 35 cycles of denaturation at 94°C for 30 seconds, annealing at 60°C for 30 seconds, and extension at 72°C for 30 seconds, and an additional extension at 72°C for 5 minutes. PCR products (10 μL) were analyzed in a 1.2% agarose (Invitrogen) gel with 3 μL ethidium bromide (10 mg/mL dissolved in water) (Sigma-Aldrich). A 100 bp molecular marker was used (GeneRuler, Thermo Fisher Scientific) to determine amplicon size. Samples were run for 30 minutes at 135 V in the electrophoresis chamber (Mupid-One, Advance). Gels were analyzed using the Bio-Rad GelDoc Go Imaging system.

### Insulin tolerance test

Mice were fasted for 6 hours (8:00 am to 2:00 pm) with free access to water. Baseline blood glucose levels were measured from the tail vein using an Accu-Chek glucometer (Roche). Human recombinant insulin (Humulin R, Eli Lilly) was administered via intraperitoneal injection at a dose of 0.5 U/kg body weight. Blood glucose levels were subsequently measured at 15, 30, 45, 60, 90, and 120 minutes after injection. For each time point, a small blood sample was collected from the tail vein using a sterile lancet. Area under the curve (AUC) was calculated using the trapezoidal method to quantify the overall glycemic response. All insulin tolerance tests were performed in sibling wild-type and *Hnf1a*^+/*Δ*e4-10^ mice at 7 weeks of age (*n =* 5–9 per group).

### Oral glucose tolerance test

After overnight fasting (16 hours) or 6-hour fasting (for 3 weeks of age), *Hnf1a^+/+^* mice and *Hnf1a*^+/*Δ*e4-10^ mice received an oral bolus of glucose (2 g/kg). Glycemia was measured at 0, 15, 30, 60, 90, and 120 minutes using an Accu-Chek glucometer (Roche) on tail blood samples.

For hormone measurements, blood samples were collected via tail vein into heparinized capillary tubes at baseline (*t*_0_) and at 15 and 30 minutes after glucose administration for insulin and glucagon measurement, respectively. After centrifugation, plasma was separated and stored at –80°C until analysis.

### Semiquantitative determination of glycosuria

Urine was collected from mice in diuresis cages after 16 hours of fasting and after oral glucose tolerance testing (OGTT). Urinary glucose concentration was determined using the Glucose Colorimetric Detection Kit (Invitrogen) according to the manufacturer’s instructions. A standard curve was generated using 8-point serial dilutions of the glucose standard (320 mg/dL): 32, 16, 8, 4, 2, 1, 0.5, and 0 mg/dL in assay buffer. Urine samples were diluted 1:2, 1:4, or 1:100 in assay buffer depending on expected glucose concentration. Standards (20 μL) or diluted samples were added to 96-well plates, followed by HRP solution (25 μL), substrate (25 μL), and glucose oxidase (25 μL). Plates were incubated for 30 minutes at room temperature, and absorbance was measured at 560 nm. Glucose concentrations were calculated from the standard curve using 4-parameter logistic regression.

### Pyruvate tolerance test

Mice were fasted for 6 hours (8:00 am to 2:00 pm) with free access to water. Baseline blood glucose was measured from the tail vein using an Accu-Chek glucometer (Roche). Sodium pyruvate (2 g/kg body weight) was administered via intraperitoneal injection, and blood glucose levels were measured at 15, 30, 60, 90, and 120 minutes after injection. AUC was calculated using the trapezoidal method to quantify hepatic gluconeogenesis capacity.

### Mouse islet isolation and culture

Pancreatic islets were isolated from mice fed standard chow diet, using collagenase digestion. Mice were euthanized by cervical dislocation under deep anesthesia. The abdomen was sterilized with 70% ethanol, and the pancreas was exposed via midline incision. Cold collagenase P solution was infused through the common bile duct to distend the pancreas, which was then excised and digested in a 37°C water bath for 8–15 minutes. Digestion was terminated by addition of cold Hanks balanced salt solution containing 1% albumin.

Islets were purified using discontinuous polysucrose density gradient centrifugation (Mediatech) at densities of 1.132, 1.108, 1.096, 1.069, and 1.000 g/mL. Isolated islets demonstrated greater than 90% purity with minimal exocrine contamination. Islets were cultured overnight (18 hours) in RPMI 1640 medium (Sigma-Aldrich) supplemented with 11 mmol/L glucose, 10% FBS, and 1% penicillin/streptomycin at 37°C with 5% CO_2_ before functional studies. For dynamic perifusion studies, 300 islet equivalents (IEQ) pooled from 7 mice were perfused with varying glucose concentrations (5.5, 16.7 mmol/L) with or without gliclazide (10 μmol/L) or triple agonist (150 nmol/L) as described in the figure legends.

### Human islet isolation and culture

Human pancreatic islets were obtained from brain-dead adult donors through established clinical islet transplantation programs (ClinicalTrials.gov NCT01123187, NCT00446264, and NCT01148680) and isolated as previously described (61). Donor characteristics are reported in Supplemental Table 1.

All human islet preparations underwent quality control assessment to confirm glucose-stimulated insulin secretion (GSIS) responsiveness prior to experimentation. Islets were cultured in a glucose-free medium (Gibco, Life Technologies) supplemented with 5.5 mmol/L glucose, 0.625% human serum albumin, and 1% penicillin/streptomycin at 37°C with 5% CO_2_.

### Human islet transfection protocol

Human islets cells were cultured in uncoated 12-well plates with CMRL complete medium before transfection. Islets (2,500 IEQ per condition) were washed once with PBS and dissociated using Accutase (PAA Laboratories) for 3 minutes total: 1 minute at room temperature, 1 minute at 37°C, and 1 minute at room temperature. Dissociation was stopped by addition of CMRL complete medium. After washing with PBS, dissociated islet cells were resuspended in 1 mL CMRL medium (without supplements) containing siRNA transfection reagent complexes.

siRNAs were resuspended in RNase-Free 1× siRNA buffer (Dharmacon) at 20 μM and stored at –20°C. For transfection, siRNAs (25 nM or 50 nM final concentration) were preincubated with DharmaFECT 4 transfection reagent (5 μL/mL; Dharmacon) for 5 minutes at room temperature according to the manufacturer’s instructions. Non-targeting scrambled control siRNA or human HNF1A ON-TARGETplus siRNA (Dharmacon) was used at a final concentration of 25 nM or 50 nM. After 16 hours, transfection medium was replaced with CMRL complete medium. Protein extraction, RNA isolation, and glucose-stimulated hormone secretion assays were performed 48 hours after transfection.

### Static incubation techniques

For glucose-stimulated glucagon secretion, islets (50 IEQ per well per quadruplicate) were preincubated for 1 hour at 37°C with 5% CO_2_ in glucose-free RPMI 1640 medium (Gibco) supplemented with 0.625% human serum albumin, 1% penicillin/streptomycin, and stimulatory glucose (10 mmol/L for human islets; 16.7 mmol/L for mouse islets).

Following preincubation, medium was replaced with either low glucose (2 mmol/L for human; 2 mmol/L for mouse) or high glucose (10 mmol/L for human; 16.7 mmol/L for mouse). After 1 hour of incubation, supernatants were collected and stored at –80°C. Islets were extracted in acid ethanol (1.5% HCl, 70% ethanol, 28.5% H_2_O) for measurement of intracellular hormone content. Insulin and glucagon concentrations in supernatants and extracts were measured by ELISA (Mercodia) or by ultrasensitive immunoassay (Beckman Coulter Access). Hormone secretion was expressed as a percentage of total intracellular hormone content and normalized to basal secretion.

### Perifusion for dynamic hormone secretion

The kinetics of insulin or glucagon secretion in response to glucose and pharmacological agents was assessed using perifusion as previously described (62). Krebs-Ringer bicarbonate (KRB) buffer contained (in mmol/L): 120 NaCl, 4.8 KCl, 2.5 CaCl_2_, 1.2 MgCl_2_, and 24 NaHCO_3_, supplemented with 0.1% BSA. Buffer was continuously gassed with O_2_/CO_2_ (94:6 vol/vol) and maintained at 37°C throughout experiments.

#### Insulin secretion protocol.

Approximately 300 IEQ were loaded into perifusion chambers and equilibrated for 50 minutes in low glucose (3 mmol/L for human islets; 5.5 mmol/L for mouse islets) without sample collection. Islets were then sequentially exposed to varying glucose concentrations (5.5, 16.7 mmol/L) with or without gliclazide (10 μmol/L) or triple agonist (150 nmol/L) as detailed in the figure legends.

#### Glucagon secretion protocol.

Islets (300 IEQ) were equilibrated for 50 minutes in basal glucose (6 mmol/L for human islets; 16.7 mmol/L for mouse islets). KRB buffer was supplemented with 2 mmol/L each of alanine, glutamine, and arginine to stimulate glucagon secretion. Islets were then exposed to low (1–2 mmol/L) and high (15 mmol/L) glucose as detailed in the figure legends.

Perifusate fractions were collected at specified intervals, and flow rate, pressure, temperature, and oxygenation were maintained constant. After perifusion, islets were extracted in acid ethanol (500 μL) and stored at –80°C for measurement of total hormone content.

### Hormone measurements

Insulin, glucagon, and proinsulin concentrations in islet supernatants, extracts, and mouse plasma were measured by ELISA or immunoassay according to the manufacturer’s instructions. Specific assay kits are detailed in Supplemental Table 4.

Islet supernatants and acid ethanol extracts were diluted in PBS containing 0.1% BSA before analysis. Plasma samples were collected from mice at fasting (*t*_0_), 15 minutes, and 30 minutes during OGTT and stored at –80°C until analysis.

### RT-qPCR analysis

Total RNA was extracted from human islets or mouse tissues using the RNeasy Mini Kit (QIAGEN). RNA concentration was measured by NanoDrop spectrophotometry. First-strand cDNA was synthesized using SuperScript IV reverse transcriptase (Life Technologies) according to the manufacturer’s instructions.

RT-qPCR was performed using the CFX Connect Real-Time System (Bio-Rad) with SYBR Green Supermix. Primers (Supplemental Table 5) were designed using Primer3 software (https://www.primer3plus.com/) and used at 500 nM final concentration (250 nM each primer). cDNA was diluted 1:10, and 2 μL was used per 10 μL reaction. Thermal cycling conditions were: 95°C for 3 minutes, followed by 40 cycles of 95°C for 10 seconds and 60°C for 30 seconds. Melt curve analysis confirmed primer specificity. Gene expression was normalized to RPL27 or RPLP0 housekeeping gene using the 2^-ΔCt^ method.

### Protein extraction

Human islets (2,000 IEQ) or mouse tissues (~100 mg) were lysed in protein lysis buffer containing (in mmol/L): 20 Tris-acetate, 270 sucrose, 1 EDTA, 1 EGTA, 50 sodium fluoride, 10 β-glycerophosphate, and 1% Triton X-100, supplemented with protease inhibitor cocktail (Sigma-Aldrich, catalog P8340) and phosphatase inhibitor (PhosSTOP, Roche). Lysis volumes were 80 μL for islets and 400 μL for tissues.

Samples were sonicated for 4 minutes in an ultrasonic water bath (UCP-02, Lab Companion), incubated on ice for 20 minutes, and centrifuged at 13,500*g* for 20 minutes at 4°C. Supernatants were collected and protein concentration was determined using the Pierce BCA Protein Assay Kit (Thermo Fisher Scientific).

### Western blot analysis

Equal amounts of protein (20–40 μg) were mixed with 4× Laemmli sample buffer (Alfa Aesar), denatured at 95°C for 5 minutes, and separated by SDS-PAGE using either 4%–12% Bis-Tris gels (Invitrogen) for high–molecular weight proteins or 20% homemade Bis-Tris gels for low–molecular weight proteins. Electrophoresis was performed at 165 V for 50 minutes in MES running buffer (Millipore).

Proteins were transferred to nitrocellulose membranes using the iBlot 2 Gel Transfer Device (Thermo Fisher Scientific) at 25 V for 6 minutes. Membranes were stained with Ponceau S to verify equal loading (Supplemental Table 6), blocked in 5% BSA in TBST for 1 hour at room temperature, and incubated overnight at 4°C with primary antibodies (Supplemental Table 6). After washing 3 times with TBST, membranes were incubated with HRP-conjugated secondary antibodies (1:10,000 in 5% BSA-TBST; Supplemental Table 7) for 1 hour at room temperature. After 3 additional TBST washes, proteins were detected using ECL Plus substrate (GE Healthcare) and imaged on an Amersham 600 system. β-Actin or GAPDH served as a loading control.

### Immunofluorescence

Mouse pancreatic tissues were fixed in 4% paraformaldehyde/PBS for 3 hours, dehydrated through graded ethanol, and embedded in paraffin. Sections (5 μm) were deparaffinized using standard xylene and ethanol gradient protocol. Heat-mediated antigen retrieval was performed according to antibody-specific requirements (Supplemental Table 7).

Sections were blocked with serum-free protein block (Dako) for 30 minutes at room temperature and incubated overnight at 4°C with primary antibodies. After washing with PBS, sections were incubated with fluorophore-conjugated secondary antibodies for 1 hour at room temperature. Nuclei were counterstained with DAPI (Thermo Fisher Scientific) for 10 minutes. Sections were mounted with Dako Fluorescence Mounting Medium.

Images were acquired using a Zeiss LSM 710 confocal microscope with an Airyscan super-resolution module (Zeiss). Quantification and processing were performed using ImageJ software (version 2.0.0-rc-43/1.50e; https://imagej.net/ij). Primary and secondary antibody details are provided in Supplemental Table 7.

### Islet embedding for immunofluorescence

Human islets (1,000 IEQ) were washed twice with PBS, fixed in 4% paraformaldehyde/PBS for 1 hour, washed again with PBS, and embedded in pre-warmed HistoGel (Thermo Fisher Scientific). HistoGel-embedded islets were processed into paraffin blocks, sectioned at 5 μm, and subjected to immunofluorescence staining as described above.

### Masson’s trichrome staining

Tissues were fixed in 4% paraformaldehyde for 24 hours, dehydrated through graded ethanol, and embedded in paraffin. Sections (5 μm) were deparaffinized, rehydrated, and stained using a Masson’s trichrome kit. Briefly, sections were stained with Harris hematoxylin (Bio Optica) for 1 minute, washed in running tap water, incubated with Ponceau fuchsin solution (Sigma-Aldrich) for 2 minutes, washed with acidified water, differentiated in phosphomolybdic-phosphotungstic acid solution (Sigma-Aldrich) for 1 minute, and counterstained with light green FCF (Alfa Aesar) for 2 minutes. Sections were dehydrated in absolute ethanol, cleared in xylene, and mounted with permanent mounting medium. Images were acquired using a NanoZoomer S60 digital slide scanner (Hamamatsu).

### Statistics

Data are expressed as means ± SEM. Sample sizes were chosen based on previous studies demonstrating statistical significance with similar endpoints. No statistical methods were used to predetermine sample sizes. All statistical analyses were performed using GraphPad Prism 10.0 (version 10.4.1). The threshold for statistical significance was set at *P* < 0.05.

Normal distribution was assessed using the Shapiro-Wilk test. Differences between 2 groups were determined using unpaired 2-tailed *t* tests. Multiple group comparisons were analyzed using 1-way or 2-way ANOVA with Tukey’s post hoc tests. For longitudinal measurements (body weight, fasting glycemia, hormone levels), 2-way ANOVA with mixed-effects analysis was used to account for within-subject correlation across time points.

For time course experiments, AUC was calculated using the trapezoidal rule with baseline correction where appropriate. Specific statistical tests for each experiment are indicated in the figure legends.

#### Perifusion experiment analysis.

Insulin or glucagon secretion was normalized to total hormone content. Values from the last 10 minutes of each glucose phase were considered representative of steady-state secretion. The stimulus index, reflecting islet responsiveness to secretagogues, was defined as the ratio of hormone secretion in the presence of drug (gliclazide or triple GLP1R agonist) to steady-state secretion at the same glucose concentration.

For perifusion experiments, mean values for each condition (glucose ± drug) were pooled from *n* = 3 mouse islet preparations (300 IEQ pooled from 7 mice per preparation; Figure 7A) or *n* = 5 human islet donors (Figure 8, E–H). Time point–specific responses to drug administration are shown in Figure 5, C and D. Statistical significance was assessed using 2-way ANOVA with Tukey’s multiple-comparison test for matched values, evaluating main effects of glucose, drug treatment, their interaction, and pairwise comparisons.

### Study approval

All animal procedures were conducted in accordance with institutional guidelines and approved by the Institutional Animal Care and Ethics Committee of the University of Lille, France (protocol CEEA no. 8651-201701160349695 v4).

Human pancreatic islets were obtained from brain-dead adult organ donors through established clinical islet transplantation programs (ClinicalTrials.gov NCT01123187, NCT00446264, and NCT01148680) with institutional review board approval of the Centre Hospitalier Universitaire de Lille (University Hospital of Lille). Written informed consent was obtained from donor families prior to organ procurement according to standard organ donation protocols.

### Data availability

All data generated or analyzed during this study are included in the published article and its supplemental information files. The source data underlying all graphs and reported means are provided as a separate Excel file titled Supporting Data Values, with individual tabs corresponding to each figure panel. Additional raw data, analytical methods, and study materials are available upon reasonable request.

## Author contributions

IL and AAM wrote the original manuscript and worked as a team on conceptualization, investigation, experimental procedures, and analysis. Co–first authorship order was determined based on IL’s primary role in the comprehensive temporal characterization across multiple age groups (3 to 70 weeks) and AAM’s foundational work establishing the model and conducting all human islet validation studies. CB and AL conceived and designed the research studies. IL, AAM, CS, MML, SD, AEK, JT, GP, and ND conducted experiments and acquired data. IL, AAM, CS, and SE analyzed data and created figures. VG, PG, JKC, FP, and AL provided reagents and resources. VG, JKC, and FP supervised human islet studies and data analysis. CB provided overall project supervision and administration. IL and CB wrote the original manuscript draft. AAM, CS, SE, PG, JKC, FP, and AL reviewed and edited the manuscript. All authors read and approved the final manuscript.

## Conflict of interest

The authors have declared that no conflict of interest exists.

## Funding support

- European Foundation for the Study of Diabetes/Novo Nordisk-2022 (NNF21SA0074506 to CB).
- European Foundation for the Study of Diabetes/Lilly-2016 (to CB).
- I-SITE ULNE PhD Stipend (to AAM, supervised by CB and AL).
- University of Lille PhD Stipend (to IL).
- European Consortium for Islet Transplantation through the Juvenile Diabetes Research Foundation (to JKC and FP).
- European Genomic Institute for Diabetes ANR-10-LABX-0046 (to FP).

## Supplementary Material

Supplemental data

Unedited blot and gel images

Supporting data values

## Version 1 05/08/2026

Electronic publication

## Version 2 05/21/2026

revised graphical abstract
