# Supplementary material for: Progressive HNF1A-MODY pathophysiology revealed by a translational mouse model
Source: JCI Insight. 2026 May 8;11(9):e198095. doi: 10.1172/jci.insight.198095 (PMC13232485; doi:10.1172/jci.insight.198095)
Supplement: Supplemental data [file jciinsight-11-198095-s308.pdf]

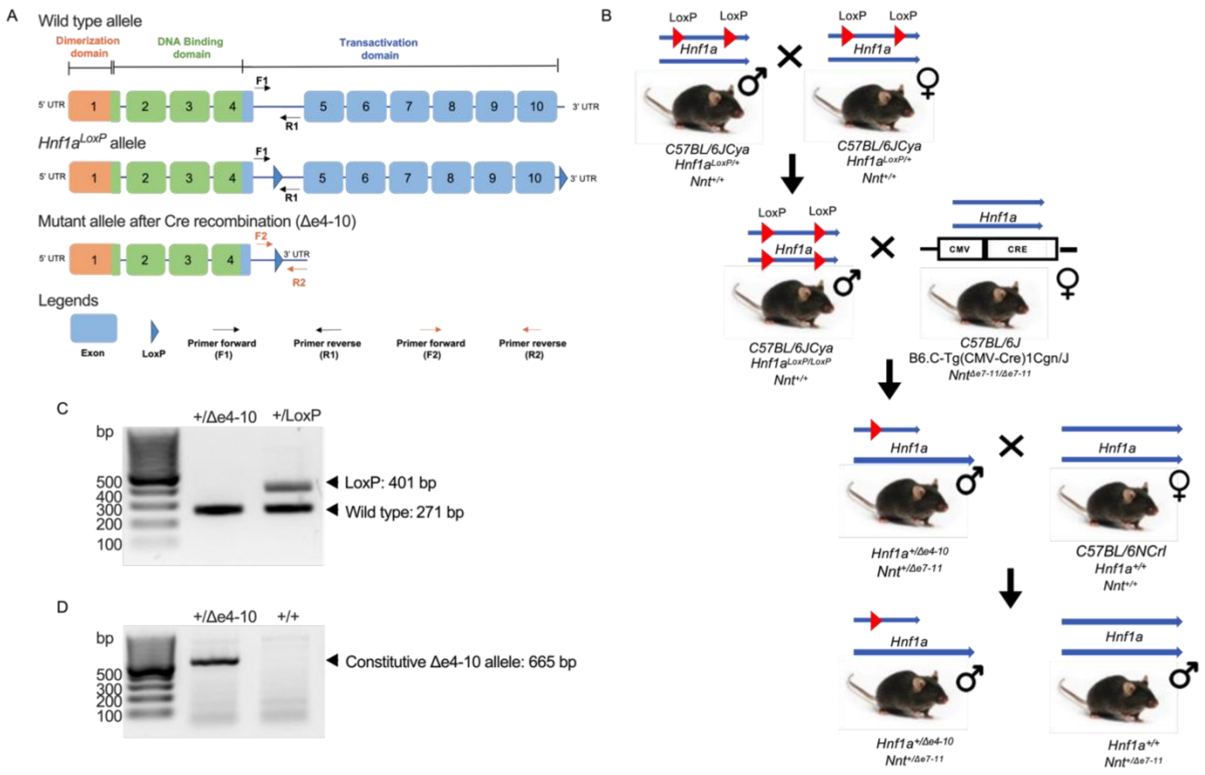

Supplemental Figure 1. Generation and validation of heterozygous *Hnf1a*$^{+/\Delta e4\text{-}10}$ mice. (**A**) Schematic representation of the *Hnf1a* gene structure showing wild-type allele, Hnf1aLoxP allele, and mutant allele after Cre recombination (Δe4-10). The gene contains exons 1-10 with dimerization domain (exons 1-2), DNA binding domain (exons 2-4), and transactivation domain (exons 5-10). Primer locations are indicated: F1/R1 primers flank the LoxP site in intron 4 to detect LoxP sites, while F2/R2 primers in exon 4 and 3'UTR detect the mutant Δe4-10 allele after deletion of exons 5-10. (**B**) Breeding strategy to generate *Hnf1a*$^{+/\Delta e4\text{-}10}$ mice. *Hnf1a*$^{\text{LoxP}/+}$ mice (C57BL/6JCya background) were bred to obtain *Hnf1a*$^{LoxP/LoxP}$ mice, then crossed with CMV-Cre transgenic mice (B6.C-Tg(CMV-Cre)1Cgn/J with *Nnt*$^{\Delta e7\text{-}11/\Delta e7\text{-}11}$) to generate *Hnf1a*$^{+/\Delta e4\text{-}10}$ mice. Final breeding was with C57BL/6NCrl mice (*Hnf1a* $^{+}/^{+}$, *Nnt* $^{+}/^{+}$) to obtain littermate controls. (**C**) PCR genotyping using F1/R1 primers showing wild-type band (271 bp) and LoxP-containing band (401 bp) to distinguish +/Δe4-10 and +/LoxP genotypes. (**D**) PCR genotyping using F2/R2 primers showing the constitutive Δe4-10 allele (665 bp) present only in +/Δe4-10 mice but absent in +/+ mice.

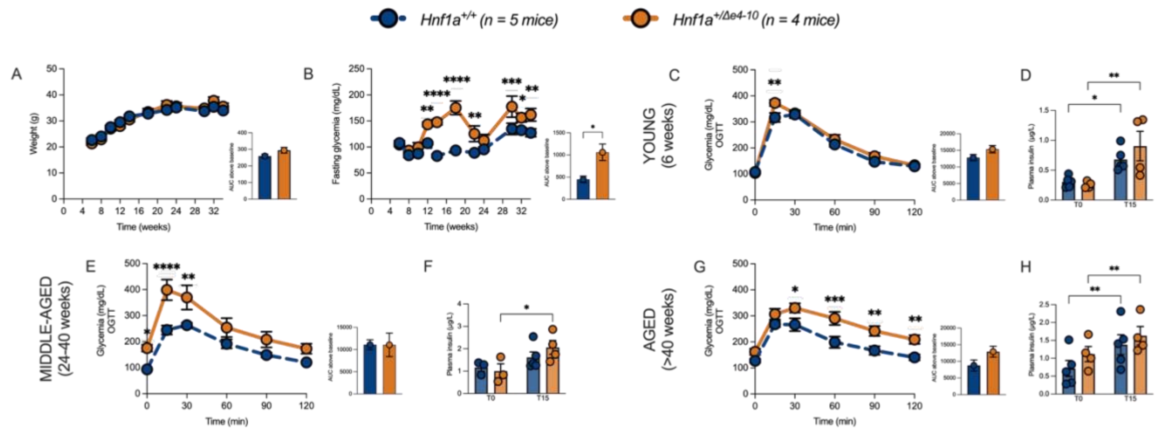

Supplemental Figure 2. Metabolic parameters in male Hnf1a+/Δe4-10 mice across different age groups. (**A**) Body weight of *Hnf1a* $^{+/+}$ mice (blue line) and *Hnf1a*$^{+/\Delta e4\text{-}10}$ mice (orange line) from 6 to 32 weeks of age. (**B**) Fasting glycemia after an overnight fast (16 h) from 6 to 32 weeks of age. Young (6 weeks): (**C**) Glycemia during OGTT (2g/kg) with area under the curve (AUC), (**D**) plasma insulin (fasting T0 and 15 min T15 after glucose challenge). Middle-aged (24-40 weeks): (**E**) Glycemia during OGTT with AUC, (**F**) plasma insulin (fasting T0 and 15 min T15 after glucose challenge). Aged (>40 weeks): (**G**) Glycemia during OGTT with AUC, (**H**) plasma insulin (fasting T0 and 15 min T15 after glucose challenge). *Hnf1a* $^{+/+}$ male mice (n = 5) and *Hnf1a*$^{+/\Delta e4\text{-}10}$ male mice (n = 4). Data are expressed as means ± SEM. *p < 0.05, **p < 0.01, ***p < 0.001, ****p < 0.0001; Two-way ANOVA with mixed-effects analysis for longitudinal data, unpaired t-test for AUC and insulin comparisons.

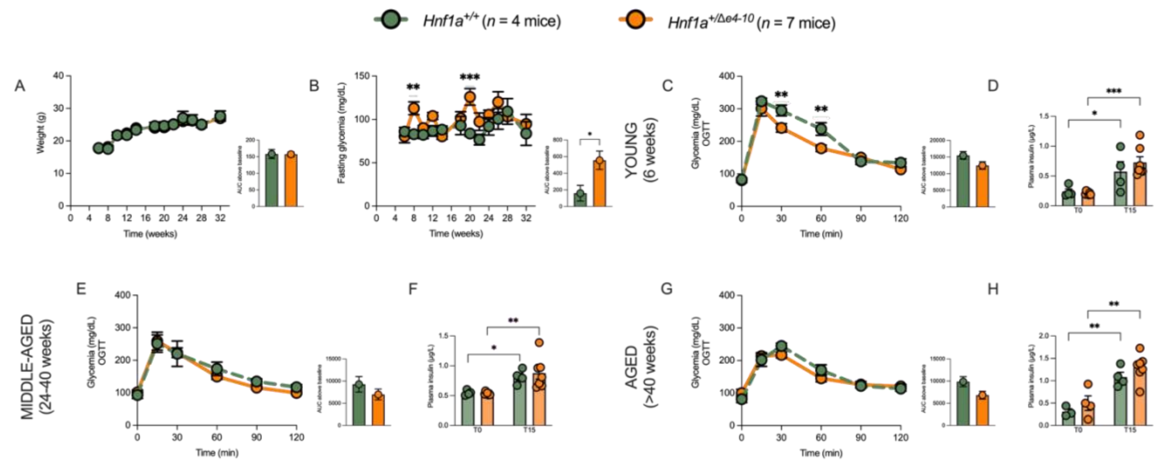

Supplemental Figure 3. Metabolic parameters in female *Hnf1a*$^{+/\Delta e4\text{-}10}$ mice. (**A**) Body weight of *Hnf1a* $^{+/+}$ mice (blue line) and *Hnf1a*$^{+/\Delta e4\text{-}10}$ mice (orange line) from 6 to 32 weeks of age. (**B**) Fasting glycemia after an overnight fast (16 h) from 6 to 32 weeks of age. Young (6 weeks): (**C**) Glycemia during OGTT (2g/kg) with area under the curve (AUC), (**D**) plasma insulin (fasting T0 and 15 min T15 after glucose challenge). Middle-aged (24-40 weeks): (**E**) Glycemia during OGTT with AUC, (**F**) plasma insulin (fasting T0 and 15 min T15 after glucose challenge). Aged (>40 weeks): (**G**) Glycemia during OGTT with AUC, (**H**) plasma insulin (fasting T0 and 15 min T15 after glucose challenge). *Hnf1a* $^{+/+}$ female mice (n = 4) and *Hnf1a*$^{+/\Delta e4\text{-}10}$ female mice (n = 7). Data are expressed as means ± SEM. *p < 0.05, **p < 0.01, ***p < 0.001; Two-way ANOVA with mixed-effects analysis for longitudinal data, unpaired t-test for AUC and insulin comparisons.

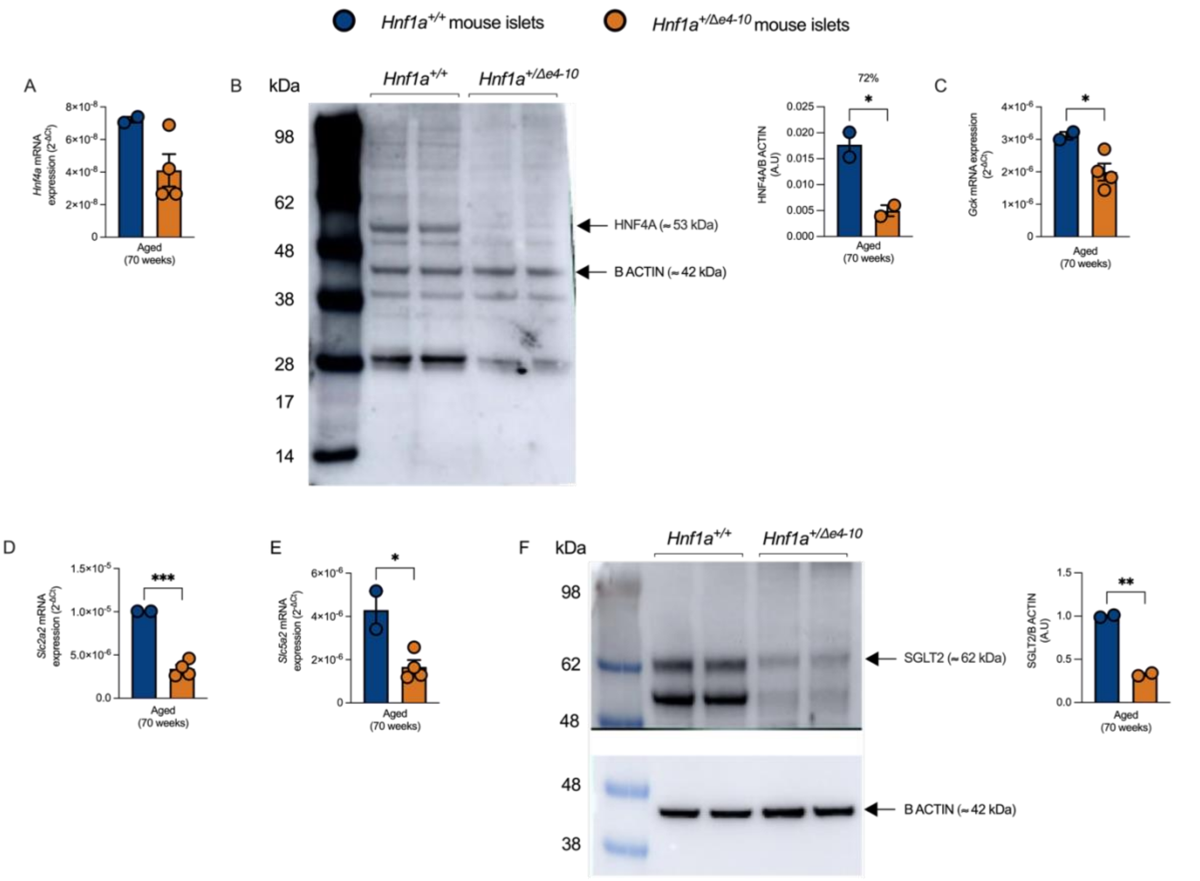

Supplemental Figure 4. Islet gene and protein expression in aged mice (70 weeks). (**A**) *Hnf4a* mRNA expression. (**B**) Representative Western blot and quantification of HNF4A protein levels. (**C**) *Gck* mRNA expression. (**D**) *Slc2a2* (GLUT2) mRNA expression. (**E**) *Slc5a2* (SGLT2) mRNA expression. (**F**) Representative Western blot and quantification of SGLT2 protein levels. Protein abundance was normalized to β-ACTIN. Data are presented as mean ± SEM. *p < 0.05, **p < 0.01, and ***p < 0.001. Unpaired t-test for qPCR and Western blot analysis.

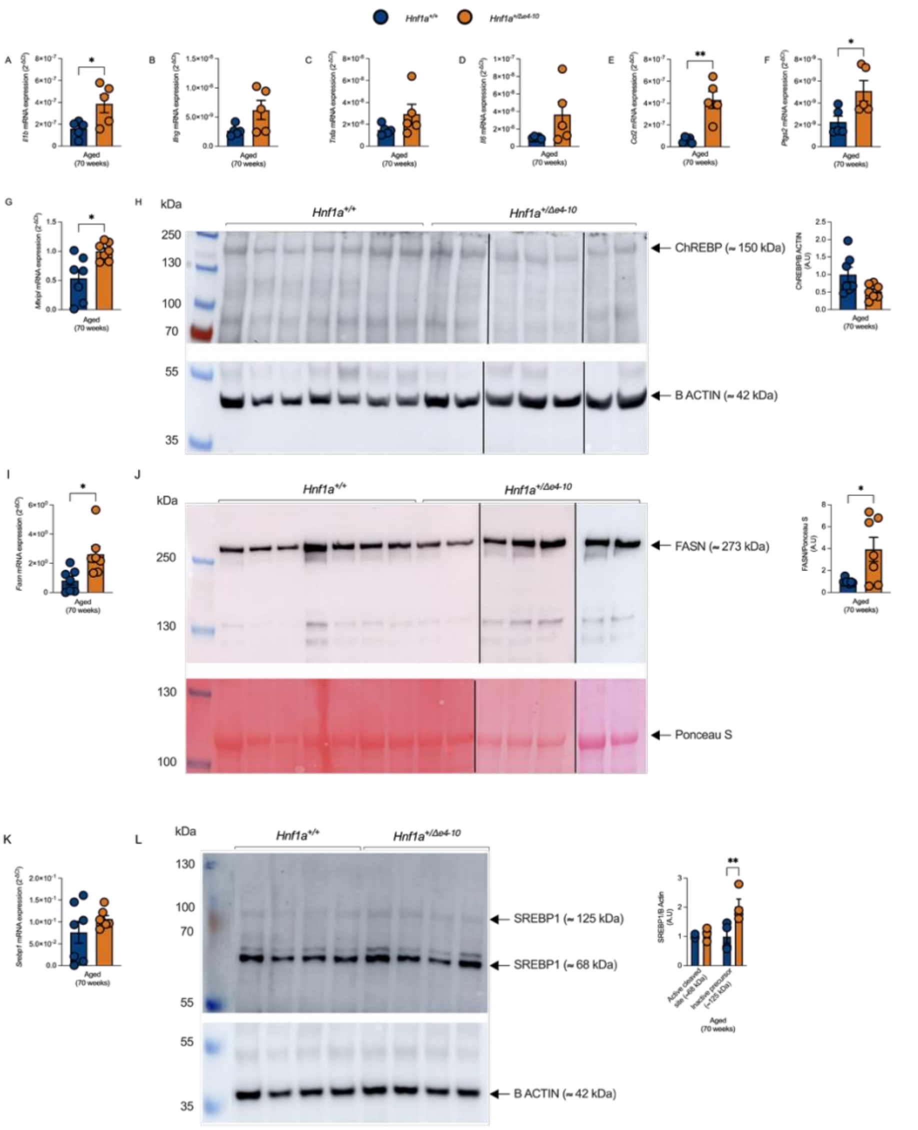

Supplemental Figure 5. Hepatic inflammatory and lipogenic profiles in aged mice (70 weeks). (**A-F**) mRNA expression of inflammatory markers: (**A**) *Il1b*, (**B**) *Ifng*, (**C**) *Tnfa*, (**D**) *Il6*, (**E**) *Ccl2*, and (**F**) *Ptgs2*. (**G**) *Mlxipl* (ChREBP) mRNA expression. (**H**) Representative Western blot and quantification of ChREBP protein (≈ 150 kDa) normalized to β-ACTIN. (**I**) *Fasn* mRNA expression. (**J**) Representative Western blot and quantification of FASN protein (≈ 273 kDa) normalized to Ponceau S. (**K**) *Srebp-1* mRNA expression. (**L**) Representative Western blot and quantification of SREBP-1 protein forms (precursor ≈ 125 kDa; active cleaved ≈ 68 kDa) normalized to β-ACTIN. Data are presented as mean ± SEM. $^{*}p < 0.05$, $^{**}p < 0.01$; unpaired t-tests were used for statistical comparisons.

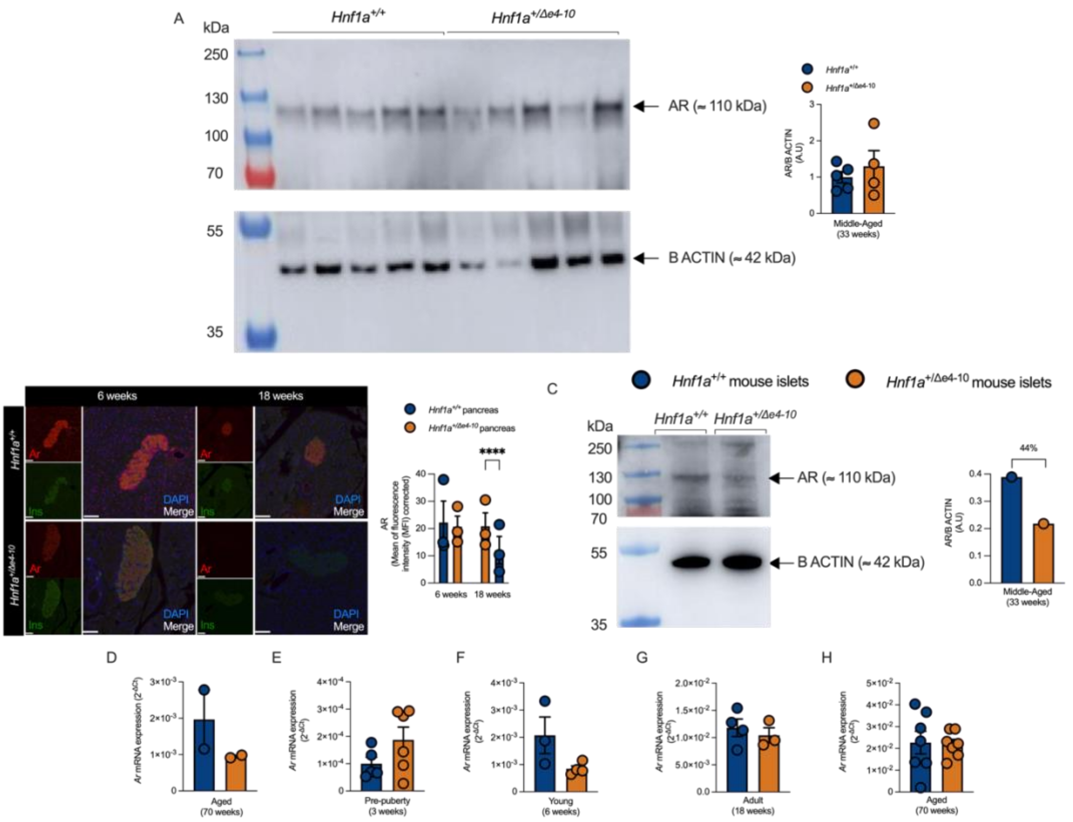

Supplemental Figure 6. Androgen receptor expression profile in testis, pancreatic tissues and liver. (**A**) Representative Western blot and quantification of AR protein (~ 110 kDa) in testis from middle-aged mice (33 weeks). B-ACTIN (~42 kDa) was used as a loading control. (**B**) Representative immunofluorescence images of pancreatic sections from young (6 weeks) and adult (18 weeks) mice stained for AR (red), Insulin (green), and DAPI (blue). The bar graph represents the quantification of mean nuclear AR fluorescence intensity (MFI) in islets. (**C**) Western blot analysis of AR protein expression (~110 kDa) in pancreatic islets from *Hnf1a* $^{+/+}$ and *Hnf1a*$^{+/\Delta e4\text{-}10}$ mice. B ACTIN (~42 kDa) was used as a loading control. **Right:** Quantification of AR protein levels normalized to B ACTIN. (**D**) *Ar* mRNA expression in isolated islets of aged mice (70 weeks). (**E-H**) Quantitative PCR analysis of *Ar* mRNA levels in the livers of (**E**) pre-pubertal mice, (**F**) young mice, (**G**) adult mice, and (**H**) aged mice (n = 3-7 mice per group). Gene expression was normalized to *Rplp0* mRNA using the $2^{-\Delta Ct}$ method. Data are presented as mean ± SEM. ****p < 0.0001.

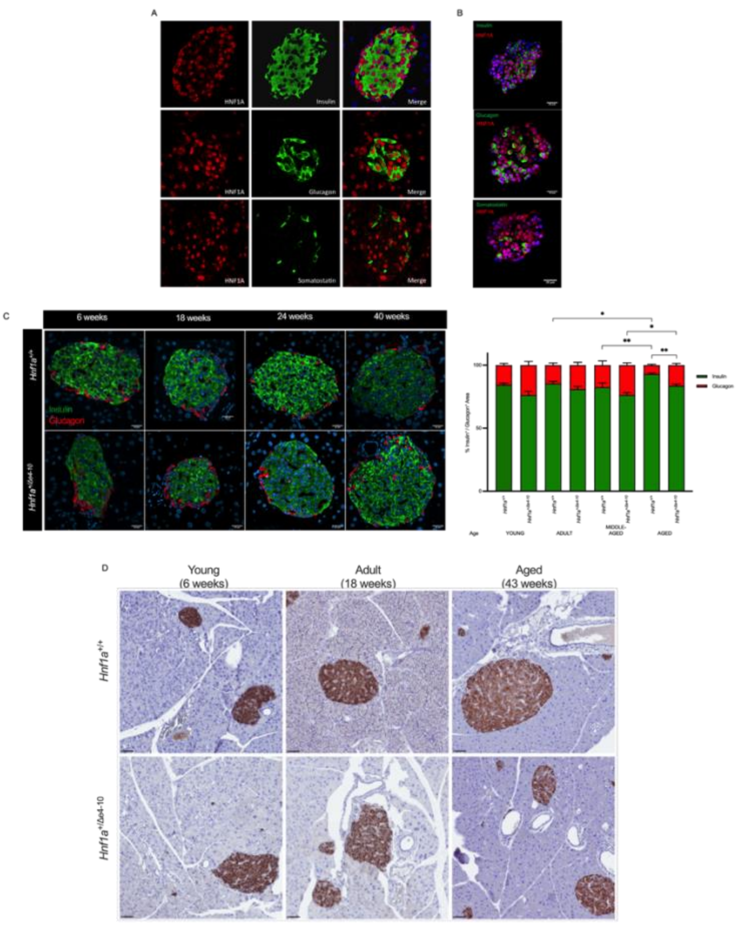

Supplemental Figure 7. HNF1A protein expression in pancreatic endocrine cells and age-dependent islet remodeling. (**A**) Representative immunofluorescence staining of mouse pancreatic sections showing HNF1A (red) localization in insulin-positive β-cells (top), glucagon-positive α-cells (middle), and somatostatin-positive δ-cells (bottom). All hormones are stained in green; nuclei are counterstained with DAPI (blue). Scale bars = 20 μm. (**B**) Representative HNF1A (red) expression in human islets co-stained with insulin, glucagon, and somatostatin (green). Nuclei counterstained with DAPI (blue). Scale bars = 20 μm. (**C**) Representative immunofluorescence images of pancreatic islets from *Hnf1a* $^{+/+}$ and *Hnf1a*$^{+/\Delta e4\text{-}10}$ mice at different ages (Young: 6 weeks; Adult: 18 weeks; Middle-aged: 24 weeks; Aged: 43 weeks) showing insulin (green) and glucagon (red) distribution. **Right panel:** Quantification of insulin-positive and glucagon-positive areas as a percentage of total islet area across age groups (Young, Adult, Middle-aged, and Aged). Data are presented as mean ± SEM. *$p < 0.05$. (**D**) Histological analysis with Masson's trichrome staining showing fat accumulation in *Hnf1a*$^{+/\Delta e4\text{-}10}$ pancreas compared to normal architecture in *Hnf1a* $^{+/+}$ mice from 6, 18 and 43 weeks. Representative images at 40X magnification; scale bars = 50 μm.

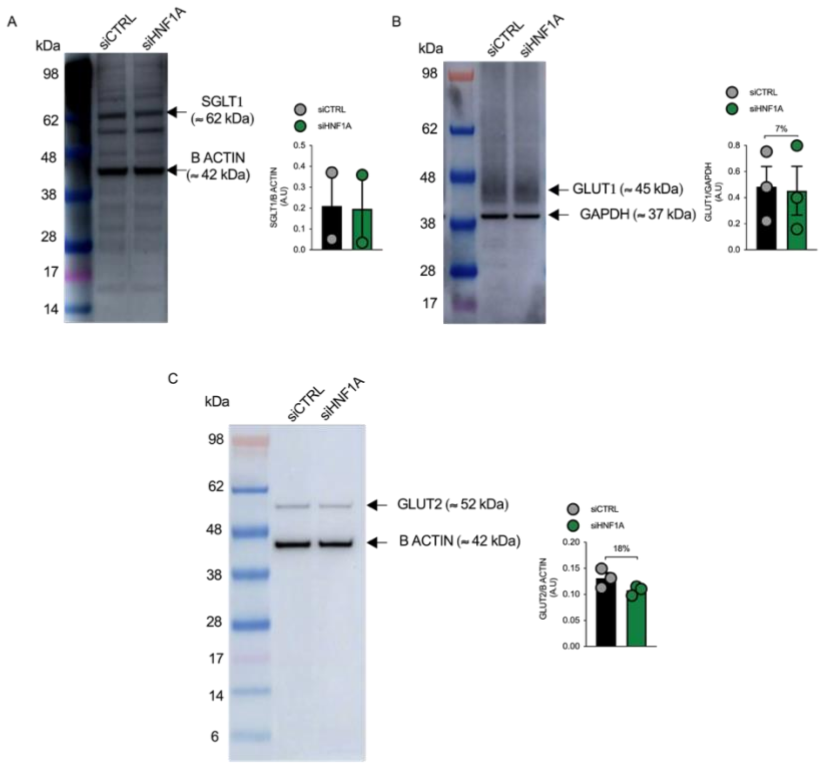

Supplemental Figure 8. Glucose transporter protein expression analysis in human islets after HNF1A knockdown. Western blot analysis of (**A**) SGLT1 (≈ 62 kDa), (**B**) GLUT1 (≈ 45 kDa), and (**C**) GLUT2 (≈ 52 kDa) protein levels in human islets transfected with control siRNA (siCTRL) or HNF1A siRNA (siHNF1A). Right panels show quantification of protein levels normalized to β-actin (≈ 42 kDa) for SGLT1 and GLUT2, and to GAPDH (≈ 37 kDa) for GLUT1. GLUT1 shows a 7% reduction and GLUT2 shows an 18% reduction after HNF1A knockdown, while SGLT1 levels remain unchanged. Protein levels were normalized to appropriate loading controls. Data are presented as means ± SEM with percentage changes indicated. Statistical analysis performed using unpaired t-tests.

Supplemental Table 1. Donor Characteristics

Donor ID	Age (years)	Sex (M/F)	BMI (kg/m^2)	HbA1c	Diabetes	Cause of death	Cold ischaemia time (h :min)	Estimated purity (%)	Estimated viability (%)	Total culture time (h)	GSIS assay	Additionnal notes
H1093	46	M	25.3	5.1	No	Choking	07 :52	90	98.8	20	1.37	*HNF1A* silencing
H1095	56	M	30.5	5.5	No	Choking	07 :22	80	98.1	23	2.77	*HNF1A* silencing
H1097	53	F	29.1	5.3	No	Traumatic stroke	03 :23	90	94.5	10	1.76	*HNF1A* silencing
H1099	54	F	24.3	5.8	No	Stroke	07 :02	90	96.2	60	4.74	*HNF1A* silencing
H1102	46	F	23.7	5.1	No	Head trauma	04 :25	80	96.3	20	4.23	*HNF1A* silencing
H1105	55	F	27.5	5.5	No	Head trauma	07 :43	80	96	20	3.69	*HNF1A* silencing
H1109	65	F	27.4	6.1	No	Stroke	06 :11	90	99	18	0.91	*HNF1A* silencing
H1114	50	M	34	5.5	No	Suicide	05 :50	95	99.2	21	2.93	*HNF1A* silencing
H1119	61	M	25.1	5.6	No	Choking	08:15	80	91.9	15	3.68	*HNF1A* silencing

Supplemental Table 2. Mouse details

GROUP Number	Number *Hnf1a*$^{+/+}$	Number *Hnf1a*$^{+/\Delta e4\text{-}10}$	Sacrifice age	Number of Backcrosses	Used for experiment
**1: pilot study males**	5	4	34 weeks	F1	• Supplemental Figure 2<br>• Figure 2K and L<br>• Figure 3G, H
**2: pilot study females**	4	7	32 weeks	F1	• Supplemental Figure 3
**3: Tissue collection**	3	4	6 weeks	F2	• Figure 1B, C, F and G<br>• Figure 3C, D<br>• Figure 4H<br>• Supplemental Figure 6B<br>• Supplemental Figure 7C and D
**4: Tissue collection**	4	4	12 weeks	F2	• Figure 3C
**5: Tissue collection**	4	3	18 weeks	F2	• Figure 3D<br>• Figure 4I<br>• Supplemental Figure 6B<br>• Supplemental Figure 7C and D
**6: Tissue collection**	4	4	24 weeks	F2	• Figure 3C<br>• Supplemental Figure 7C
**7: Longitudinal study and Tissue collection**	5	8	70 weeks	F1	• Figure 1D.<br>• Figure 2A, B, E, F, G, H, I, J, M, N, and O.<br>• Supplemental Figure 4.<br>• Figure 3B, C, I, J, K, L, M and N<br>• Supplemental Figure 5<br>• Figure 4J<br>• Supplemental Figure 6C<br>• Figure 5A, B, C and D
**8: Longitudinal study and Tissue collection**	5	5-9	43 weeks	F1	• Figure 2D<br>• Figure 3A, B, C<br>• Supplemental Figure 7C and D
**9: Longitudinal study and Tissue collection**	5	5	55 weeks	F2	• Figure 2V<br>• Figure 3C<br>• Figure 4B, C, D and E
**10: Longitudinal study and Tissue collection**	6	6	33 weeks	F3	• Figure 1H<br>• Figure 2U<br>• Figure 4F<br>• Supplemental Figure 6A
**11: Complementary study**	6	9		F4	• Figure 2P, Q, R, and S
**12: Revision study**	5	7	3 weeks	F4	• Figure 2C, T<br>• Figure 3E, F<br>• Figure 4A, G
**Total:**	**126**				

Supplemental Table 3. Primers for genotyping

**Mouse line**	**Forward sequence (5'-3')**	**Reverse sequence (5'-3')**
CMV-Cre	gcggtctggcagtaaaaactatc	gtgaaacagcattgctgtcactt
Hnf1a$^{LoxP/LoxP}$ (Neo-del cassette)<br>F1-R1	ccgtaacaagacctgatttcctt	ggctcagagaactaaacggaaatg
Constitutive Δe4-10 allele<br>F2-R2	tggacacttacactgtattggctg	tattctaaacagcagggatggtgg
Hnf1a$^{LoxP/LoxP}$::CMV-Cre<br>F3-R3-R3.1	aatcccgatgtggacacttac	aatggaaccgagccttacag<br>ccactcctgtcccaagcatt

Supplemental Table 4. Hormone Measurement kits

Samples	Hormone	Glucose stimulated hormone secretion	Hormone measurement	Dilutions
Human islets	Insulin	Perifusion technique	Immunoassay system	Supernatant :<br>No diluted<br>Content :<br>1/2114<br>1/3844<br>1/5761
Human islets	Glucagon	Perifusion technique	Glucagon ELISA kit Mercodia<br>Cat No 10-1271-01	Supernatant :<br>No diluted<br>Content :<br>1/500<br>1/1000<br>1/2000
Human islets	Glucagon	Static incubation technique	Glucagon ELISA kit Mercodia<br>Cat No 10-1271-01	Supernatant :<br>1/50<br>Content :<br>1/500
Mouse islets	Insulin	Perifusion technique	Insulin ELISA kit Mercodia<br>Cat No 10-1247-01	Supernatant :<br>No diluted<br>Content :<br>1/500<br>1/1000<br>1/2000
Mouse islets	Glucagon	Static incubation technique	Glucagon ELISA kit Mercodia<br>Cat No 10-1281-01	Supernatant :<br>No diluted<br>Content :<br>1/500
Mouse serum	Insulin	Fasting and during an OGTT	Insulin ultrasensitive ELISA kit<br>Mercodia<br>Cat No 10-1249-01	No dilution
Mouse serum	Glucagon	Fasting and during an OGTT	Glucagon ELISA kit Mercodia<br>Cat No 10-1281-01	No dilution
Mouse serum	Proinsulin	Fasting and during an OGTT	Rat/Mouse Proinsulin ELISA kit<br>Mercodia<br>Cat No 10-1232-01	No dilution
Mouse serum	Testosterone	Fasting	Testosterone rat/mouse ELISA<br>Demeditec<br>Cat No DEV9911	No dilution

Supplemental Table 5. Primers for qPCR

Species	Gene	Forward sequence (5' – 3')	Reverse sequence (5' – 3')
Human	*HNF1A*	acctgtgcagagccatgtga	ttggtggtgtcggtgatgag
	RPL27	tctggtggctggaattgacc	ccttgtgggcattaggtgattg
Mouse	*Hnf1a exon 1*	agaaacgcgtggctctgaag	ggatgttgtgctgctgcaag
	Hnf1a exon 10	cctggtgttgtatcagagttc	gccatctgggtggagata
	Hnf4a	ggcatggatatggccgacta	tcttcctcacgctcctcctg
	Slc5a1	gtatggtgtggtggccgatt	gcagatactccggcatcgtc
	Slc5a2	ggtattcatcgtggcggtgt	gcccgagccaaagaagaact
	Slc2a1	atggatcccagcagcaagaa	gcggtggttccatgtttgat
	Slc2a2	attgcggacttccttgggcc	tcctggtcggttcctcgg
	Mlxipl	ggctgactccctcttcagca	ggtggggaatggaaggagag
	Srebp-1	acgcctgtgaagggttactc	tgaccggaacacatcgactg
	Fasn	agatggaaggctgggctcta	cctctgaaccactcacaccc
	Gck	gcactgcggagatctcttt	tcggagaagtcccacgatgt
	Ar	ggcggtcattcagtattcc	ccaagtcaggtgcaaagtag
	Rplp0	ccacactgctgaacatgctg	ccctccagaaagcgagagtg
	Rpl27	tggaattgaccgctatcccc	gtgggcatgaggtggttgta
	Il1b	gtgtgtgacgttcccattag	tgtccattgaggtggagag
	Ifng	ctcttcctcatggctgtttc	ccacatctatgccacttgag
	Tnfa	cctatgtctcagcctcttct	gggaacttctcatccctttg
	Il6	agttgccttcttgggactga	tccacgatttcccagagaac
	Ccl2	aggaatgggtccagacat	ctacagaagtgcttgaggtg
	Ptgs2	gggtgtgaagggaaataagg	agtgctgggcaaagaatg

Supplemental Table 6. Ponceau Red Staining

<table>
<tr><th>Figure 1H</th><th>Supplemental Figure 6A</th><th>Data not shown (no signal)</th></tr>
<tr>
<td>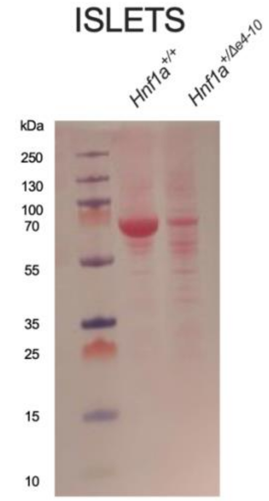
</td>
<td>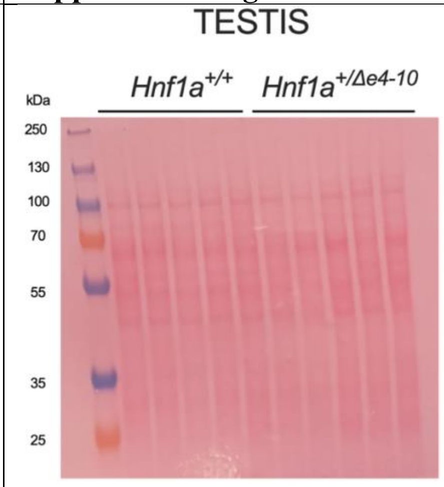
</td>
<td>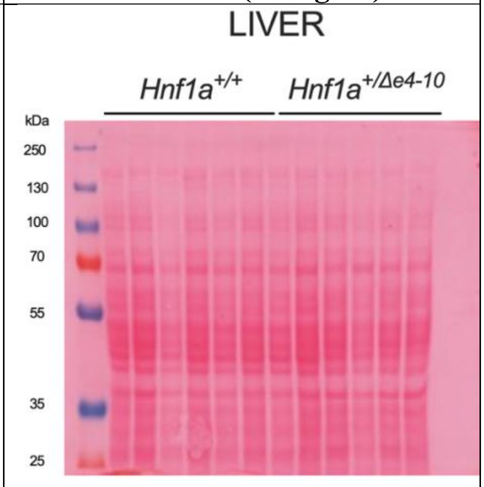
</td>
</tr>
</table>

Supplemental Table 7. Antibodies for IF and WB

Antibody	Manufacturer	Catalogue number	Species /Type	Technique	Dilution	Antigen Retrieval/Preincubation
Anti-HNF1A	Abcam	ab96777	Rabbit /Polyclonal	IF	1:100	Citrate buffer pH: 6. Waterbath 95°C. Time: 45 minutes
				WB	1:1000	N/A
Anti-AR	Abcam	ab108341	Rabbit /Monoclonal	WB	1:1000	N/A
	Merck Milipore	06-680	Rabbit/ Polyclonal	IF	1:100	Citrate buffer pH: 6. Waterbath 95°C. Time: 45 minutes
Anti-Insulin	Abcam	ab181547	Rabbit /Monoclonal	WB	1:5000	N/A
	Dako	A0564	Guinea Pig /Polyclonal	IF	1:500	Citrate buffer pH: 6. Waterbath 95°C. Time: 45 minutes
Anti-SGLT2	Novus Biologicals	NBP1-92384	Rabbit /Polyclonal	WB	1:1000	N/A
Anti-Glucagon	Abcam	ab92517	Rabbit /Monoclonal	WB	1:5000	N/A
	Gentex	GTX10988	Mouse /Monoclonal	IF	1:1000	Citrate buffer pH: 6. Waterbath 95°C. Time: 45 minutes
Anti-Somatostatin	Merck Milipore	MAB354	Rat /Polyclonal	IF	1:500	Citrate buffer pH: 6. Waterbath 95°C. Time: 45 minutes
Anti-SGLT1	Merck Milipore	07-1417	Rabbit /Polyclonal	WB	1:1000	N/A
Anti-GLUT2	Novus Biologicals	NBP2-22218	Rabbit /Polyclonal	WB	1:1000	N/A
Anti-GLUT1	Invitrogen	PA5-32428	Rabbit /Polyclonal	WB	1:1000	N/A
Anti-HNF4A	Abcam	ab201460	Rabbit /Monoclonal	WB	1:5000	N/A
Anti-CHREBP	Novus Biologicals	NB400135	Rabbit/ Polyclonal	WB	1:1000	N/A
Anti-SREBP-1	Abcam	ab28481	Rabbit/ Polyclonal	WB	1:1000	N/A
Anti-FASN	Abcam	ab128870	Rabbit/ Monoclonal	WB	1:1000	N/A
Anti-β Actin	Sigma	A5441	Mouse /Monoclonal	WB	1:5000	N/A
Anti-GAPDH	Sigma	G9545-100UL	Rabbit /Polyclonal	WB	1:5000	N/A
Anti-mouse Alexa Fluor 594	Invitrogen	A11032	N/A	IF	1:800	N/A
Anti-rabbit Alexa Fluor 594	Invitrogen	A11012	N/A	IF	1:800	N/A
Anti-rabbit Alexa Fluor 488	Invitrogen	A21206	N/A	IF	1:800	N/A
Anti-Guinea pig Alexa Fluor 488	Abcam	ab150185	N/A	IF	1:800	N/A
Anti-mouse HRP	Amersham	NXA931V	N/A	WB	1:10000	N/A
Anti-rabbit HRP	Amersham	NA934-1ML	N/A	WB	1:10000	N/A
