# Supplementary material for: Progressive HNF1A-MODY pathophysiology revealed by a translational mouse model
Source: JCI Insight. 2026 May 8;11(9):e198095. doi: 10.1172/jci.insight.198095 (PMC13232485; doi:10.1172/jci.insight.198095)

Full unedited gel for Figure 1F

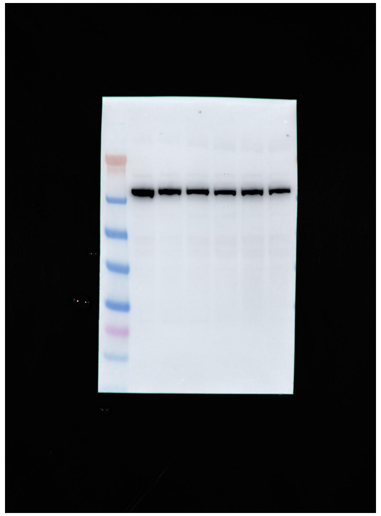

Full unedited gel for Figure 1G

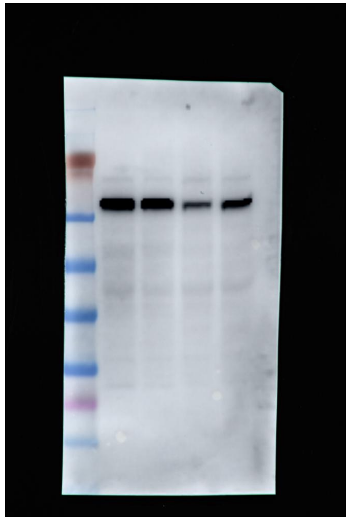

Full unedited gel for Figure 1H

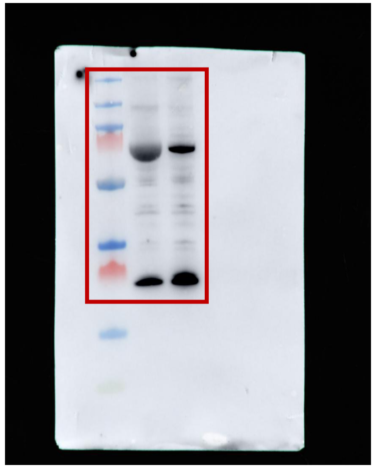

β actin full bot Fig 1F

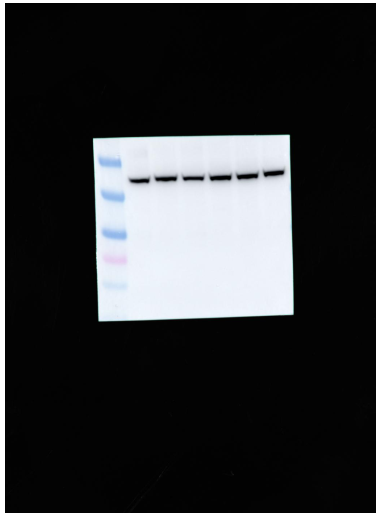

β actin full bot Fig 1G

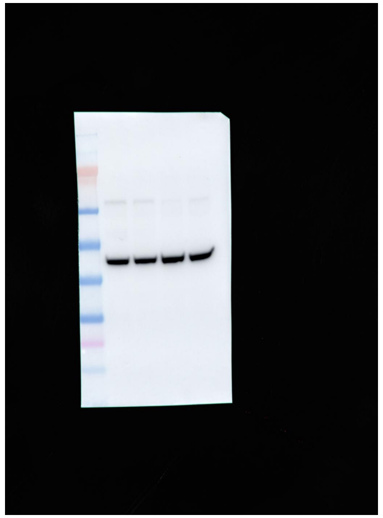

β actin full bot Fig 1H

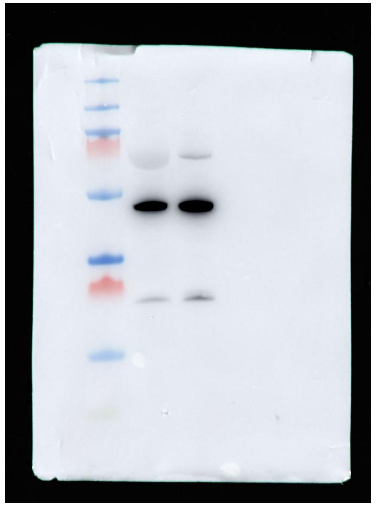

Full unedited gel for Figure 8C

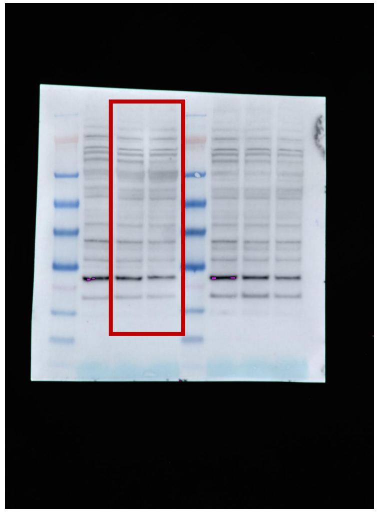

8D

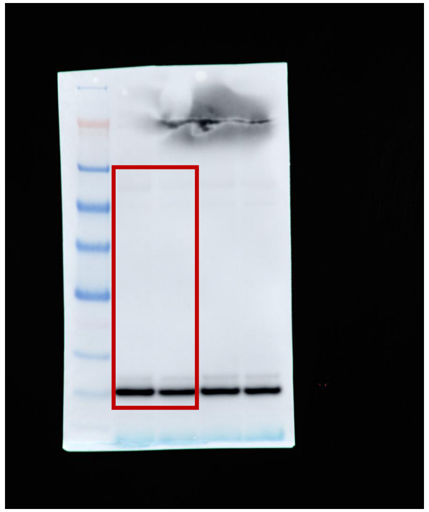

8F

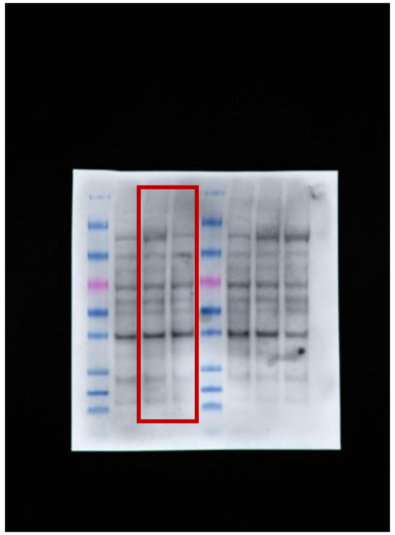

8G

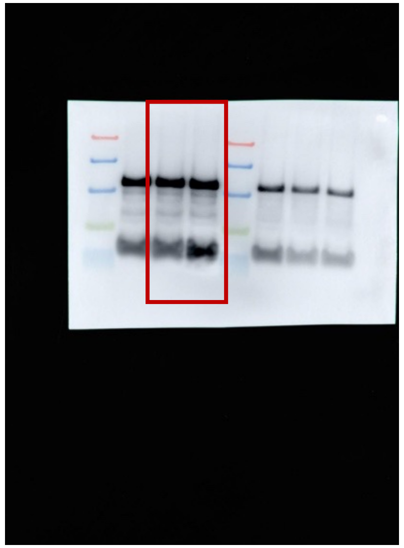

β actin full bot Fig 8C

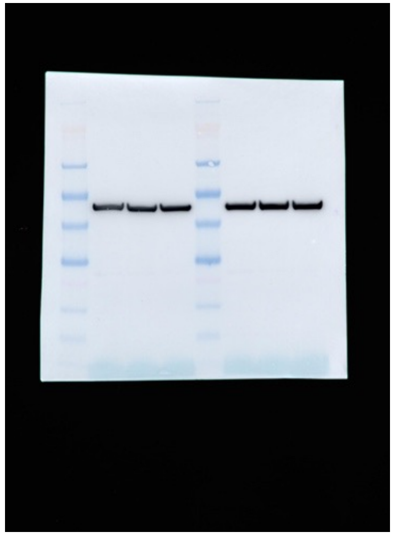

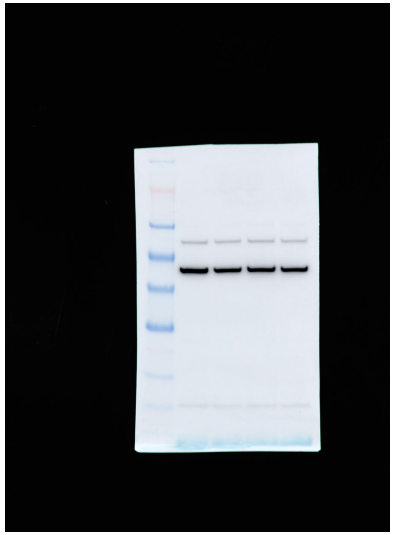

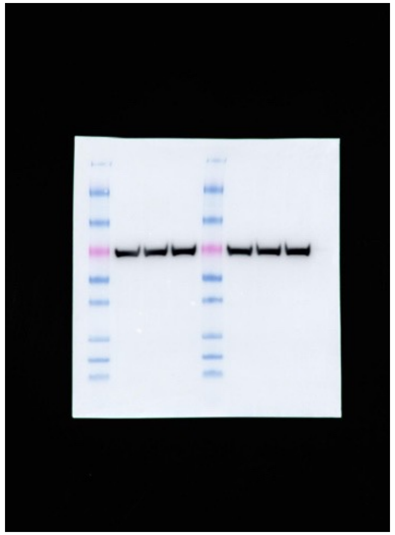

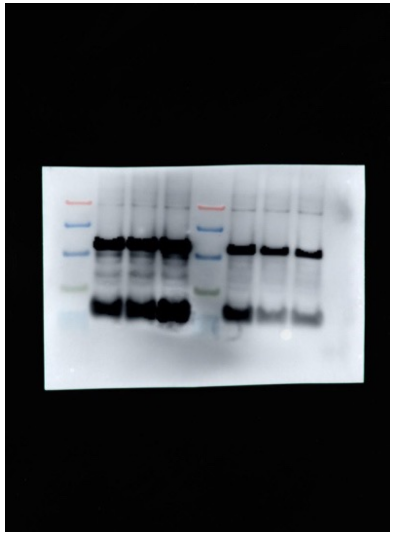

Full unedited gel for Supplemental Figure 5H

CHREBP

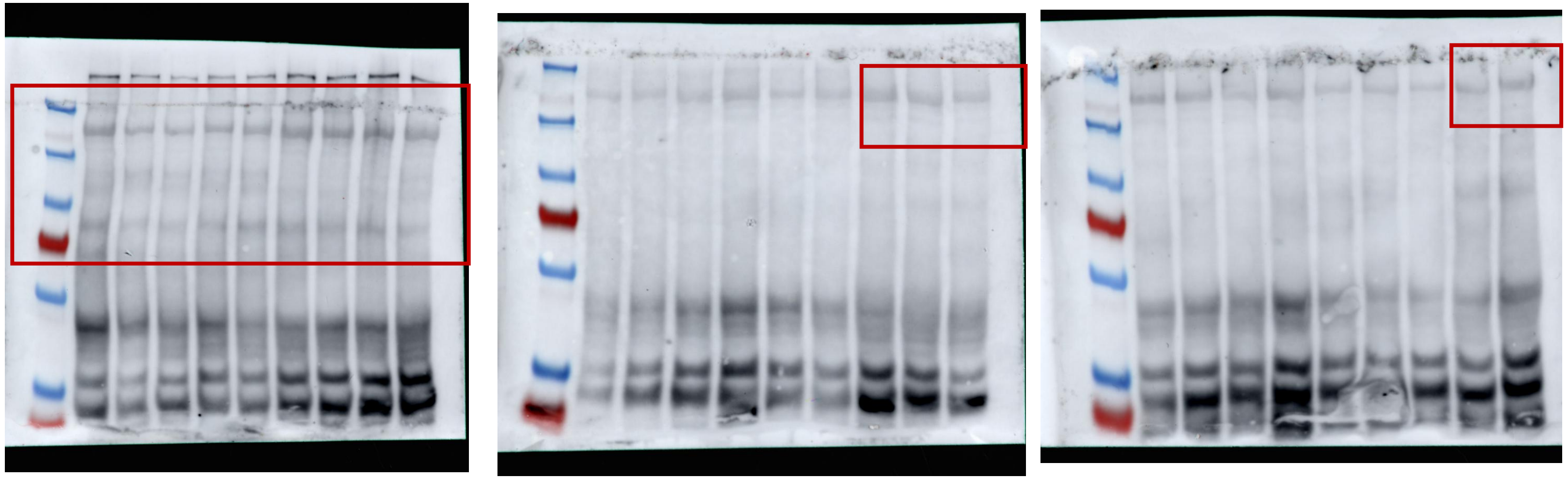

Full unedited gel for Supplemental Figure 6A

Androgen receptor

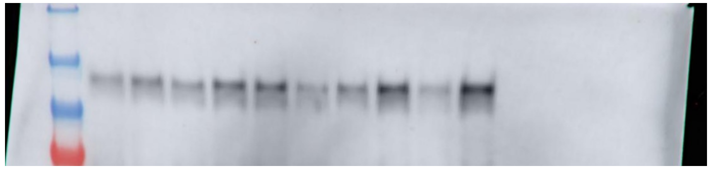

Full unedited gel for Supplemental Figure 5J

FASN

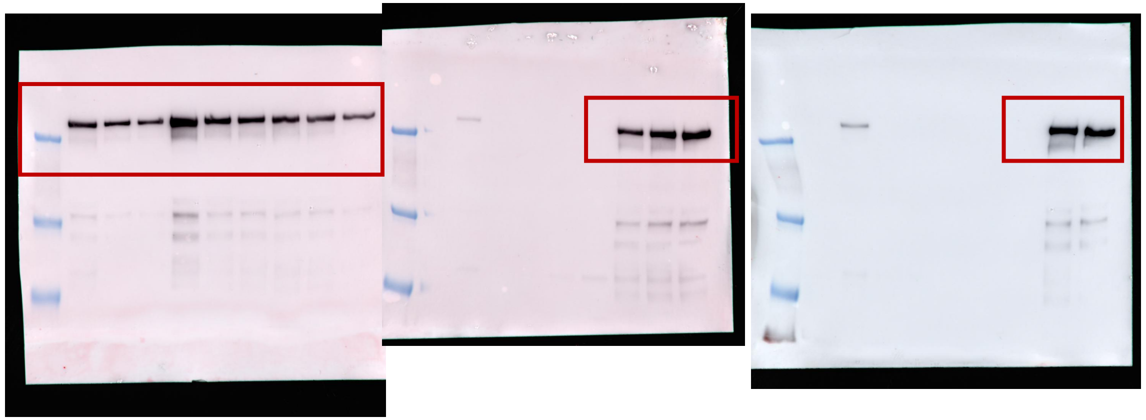

Full unedited gel for Supplemental Figure 5L

SREBP-1

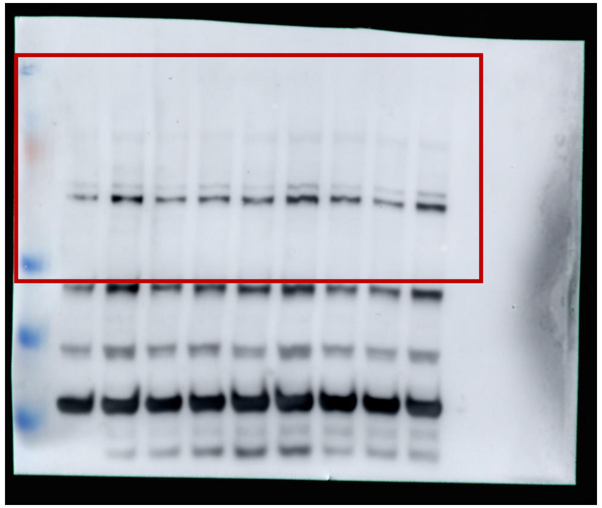

Full unedited gel for Supplemental Figure 6C

Androgen receptor

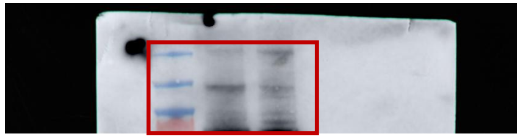

Full unedited gel for Supplemental Figure 4F

SGLT2

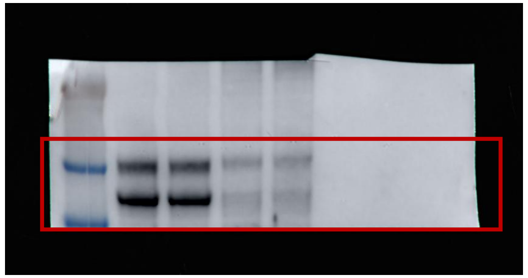

Supplement: Unedited blot and gel images [file jciinsight-11-198095-s309.pdf]
